# Monitoring Frequencies for On-Site Water Reuse: A Risk-Based Framework Applied to Greywater Reuse

**DOI:** 10.1021/acsestwater.5c01511

**Published:** 2026-04-22

**Authors:** Eva Reynaert, Michael A. Jahne, Émile Sylvestre

**Affiliations:** † 28499 Technische Universität Berlin, Water Treatment, Berlin 10623, Germany; ‡ German Environment Agency, Section II 3.3 (Water Treatment), Berlin 12307, Germany; § Office of Research and Development, U.S. Environmental Protection Agency, 26 W. Martin Luther King Drive, Cincinnati, Ohio 45268, United States; ∥ Delft University of Technology, Sanitary Engineering, Delft 2628 CN, The Netherlands; ⊥ KWR Water Research Institute, Nieuwegein 3433 PE, The Netherlands

**Keywords:** QMRA, water reuse, log-removal value, risk assessment, online monitoring

## Abstract

On-site water reuse can provide water for nonpotable applications, but ensuring long-term performance and managing treatment failures is challenging without dedicated monitoring personnel. This study proposes a risk-based framework to determine enteric pathogen log-removal targets (LRTs) as a function of operational monitoring frequency. The framework integrates (i) quantitative microbial risk assessment, (ii) modeled pathogen concentrations at three collection scales, and (iii) failure models for three treatment configurations. As an example, LRTs were calculated considering different monitoring frequencies for greywater reuse. Results show that smaller systems require less frequent monitoring due to lower pathogen occurrence compared to larger systems, e.g., >1 day at a 5-people scale vs <500 s for a 1000-people system to meet norovirus risk with a bimodal treatment barrier failing up to four times per year. Incorporating a residual disinfectant or multiple barriers extends the required monitoring intervals. While LRTs are comparable across collection scales, this study highlights a key advantage of small systems—reduced monitoring requirements—contrasting prior work that found no benefits of downsizing in terms of treatment train design. This framework can support technology developers in quantifying trade-offs between treatment and monitoring and aid regulators in establishing monitoring requirements for on-site water reuse.

## Graphical abstract

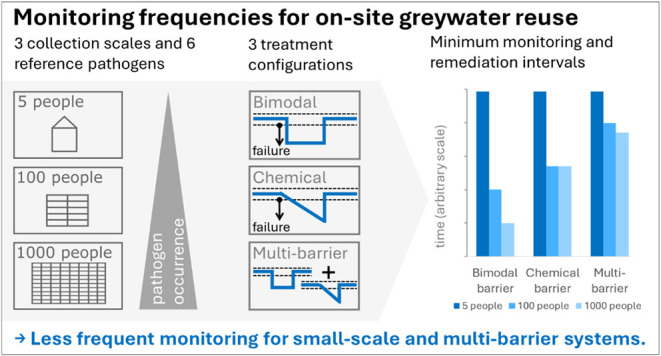

## 1 Introduction

On-site water reuse can provide consistent and predictable quantities of water for nonpotable applications without the need for large-scale infrastructure to collect the wastewater and distribute the reclaimed water. However, treatment must be in place to remove and inactivate enteric pathogens from the wastewater and prevent the regrowth of opportunistic pathogens in the treated water. The ability of a water reuse system to achieve specified pathogen reduction targets is typically validated before system approval using pathogen or microbial indicator measurements. Verification monitoring then confirms that the treatment process continues to achieve the validated reduction targets during operation, often using surrogate sensor measurements. In larger water reuse systems, e.g., at municipal scale, regular staff oversight and extensive real-time monitoring ensure treatment reliability. A key challenge for on-site water reuse is determining how and how frequently to monitor treatment performance and address treatment failures to protect user safety in the absence of personnel for operation and costly monitoring equipment.

Locally collected wastewater is characterized by highly variable pathogen concentrations.^1^ The smaller the collection scale, the lower the occurrence of enteric pathogens in the wastewater, due to a lower number of total infections within the population, but the higher the mean concentration when occurring, due to less dilution by the noninfected part of the population.^2^ Previous studies have employed quantitative microbial risk assessment (QMRA) to calculate enteric pathogen log_10_-reduction targets (LRTs) required to meet health-based targets for a range of applications of reclaimed wastewater, including indoor uses of reclaimed water, such as toilet flushing, laundry, and irrigation.^3–11^

Practical evidence from several studies suggests that on-site systems can temporarily fail in long-term operation.^12–16^ One strategy to account for treatment failures is to increase the LRTs during nominal operation to ensure that the system’s average performance, including treatment disruptions, consistently achieves the health-based targets.^17,18^ Without such additional treatment capacity, treatment failures could result in risks exceeding the health benchmark used to set the LRTs. However, none of the previously published LRTs for on-site reuse systems explicitly consider the breakthrough of pathogens during treatment disruptions.

The impact of a treatment failure on human health risks depends on both the failure mode and the effectiveness of detecting and remediating it. Several studies have developed probabilistic models to evaluate the impacts of different types of treatment failures on the overall treatment performance of a system. These models can be used to inform strategies for monitoring the microbial removal performance of a treatment process during operation, including the definition of risk-based operational monitoring frequencies for water treatment systems. Teunis and Havelaar^19^ and Teunis et al.^20^ developed a process model to calculate the average probability of passage of one or multiple treatment barriers with bimodal performance. The performance of a bimodal treatment barrier corresponds to either of two fixed values, representing nominal or failing mode of operation. Smeets et al.^21^ applied this model to calculate operational monitoring frequencies as a function of the nominal log-removal values (LRVs) of bimodal treatment processes. Sylvestre et al.^22^ expanded this work to multiple barriers in series, and developed a model for the failure of a dosing pump for chemical disinfection. Finally, Pecson et al.^23^ implemented the bimodal failure model into a probabilistic QMRA to evaluate the impact of different failure durations on risk estimates. The same study also incorporated the variability in pathogen concentrations through repeated random sampling from distributions of pathogen concentrations based on measured data from centralized wastewater treatment plants. However, this approach does not capture the intermittent presence of pathogens that characterize on-site water reuse systems, and the practical implications for operational monitoring and failure management differ from large-scale-centralized systems.

A QMRA model that integrates pathogen intermittency and treatment failures—including both failure mode and duration—can improve LRT estimates by reflecting real-world operation and use of on-site water reuse systems. We use greywater reuse for indoor nonpotable applications as an example to explore the effect of treatment failures on required LRTs and to discuss the practical implications on system design and monitoring. Pathogen intermittency is incorporated through the scale of the collection system, ranging from 5 people to 1000 people, using an epidemiology-based model that accounts for their stochastic occurrence among small populations.^2^ QMRA assumptions are closely aligned with previous studies^7,11^ regarding the selection of reference pathogens, pathogen concentrations, dose–response models, and baseline exposure scenarios, allowing the study to focus on the novel contribution of this work: developing a framework to evaluate the risk-based relationship between monitoring frequencies and LRTs in on-site water reuse. This framework enables evaluation of several trade-offs, such as the choice between single-household or building-scale reuse systems, the incorporation of single or multiple treatment barriers, and the balance between increased monitoring and stricter nominal LRTs, thus informing the design of practical, cost-effective systems that maintain reliable levels of public health protection.

## 2 Methodology

The aim of the QMRA model developed in this study was to link treatment technology failure modes and operational monitoring frequencies with the LRTs required for on-site water reuse systems to meet a health benchmark of 10^–4^ infections per person per year (pppy), allowing comparison with previous studies using the same benchmark.^7,11^ Operational monitoring intervals were defined as the time required to detect and respond to a treatment failure. On-site greywater reuse for indoor nonpotable applications was used as an application example. To evaluate the impact of varying pathogen occurrences, we examined three scales of on-site greywater reuse, notably systems that collect water from 5 people, 100 people, and 1000 people, in alignment with previous studies.^7,11^ Additionally, we explored the influence of different greywater treatment technologies by assembling treatment trains composed of one or two unit processes with different failure modes.

The determination of monitoring frequencies was based on three key principles: (1) that including additional log-removal capacities within the same unit treatment is feasible, (2) that sensors capable of reliably detecting failures of the treatment barriers at the required monitoring frequency exist, and (3) that reclaimed water is no longer consumed upon detection of a failure, e.g., by diverting off-spec water. While these principles simplify the modeling approach, making it more widely applicable and generalizable, their validity for real-world systems is discussed in Section 3.5.

Note that the epidemiology-based model to simulate reference pathogen concentrations in greywater and the main QMRA assumptions for baseline exposure to reclaimed water—including the selection of reference pathogens, dose–response models, and exposure scenarios—have been previously published and discussed. Several of these assumptions carry high uncertainty or are context-specific. Assumptions in this study are closely aligned with prior works to focus the analysis on its novel contribution, namely, the incorporation of treatment failure detection into QMRA for on-site water reuse systems. Model results can be adapted to specific cases or updated as more reliable data become available.

### 2.1 Epidemiology-Based Model to Simulate Reference Pathogen Concentrations

The QMRA incorporated six reference pathogens (*Giardia* spp., *Cryptosporidium* spp., *Campylobacter* spp., *Salmonella* spp., norovirus, and adenovirus) that were selected according to Reynaert et al.^7^ and represent pathogens linked to the highest number of gastrointestinal illnesses in the USA.

Due to the limited availability of measured concentrations of these pathogens in greywater, especially at small scales, we simulated pathogen concentrations following an epidemiology-based approach developed by Jahne et al.,^2^ with *E. coli* distributions from Sylvestre et al.^24^ To capture variability of pathogen concentrations, concentrations were calculated for each day of 10,000 possible years using a Monte Carlo approach. In short, the epidemiology-based model consists of three steps:

1) Simulating total daily infections *N* of each reference pathogen in a selected population size over 10,000 years based on generalized incidence rates and infection durations reported in the literature.
2) Simulating the daily mass concentration of feces in greywater over 10,000 years based on the distribution of measured *E. coli* concentrations in greywater, *C*_*EC,GW*_, relative to the density of *E. coli* in feces, *C*_*EC*,*F*_.
3) Inferring pathogen contributions to greywater by summing up fecal contributions from the infected part of the population and combining with the densities of pathogens shed in feces.

These steps result in the final equation for pathogen concentrations in greywater:

1 $$C_{P,GW}=\frac{1}{pop}\overset{N}{\underset{i=1}{\sum }}\frac{C_{P,F}\times C_{EC,GW}}{C_{EC,F}}$$

where dividing by the population size *pop* accounts for dilution effects by wastewater from noninfected individuals.

This approach generated a data set of 365 × 10,000 daily concentrations in greywater for each pathogen and population size (Figure 1), which was used as a QMRA model input. The full model description and distributions of all input parameters are summarized in (Supporting Information SI) Section 1, and the code used to generate pathogen concentrations is available at doi:10.25678/000GSK.

**Figure 1:**
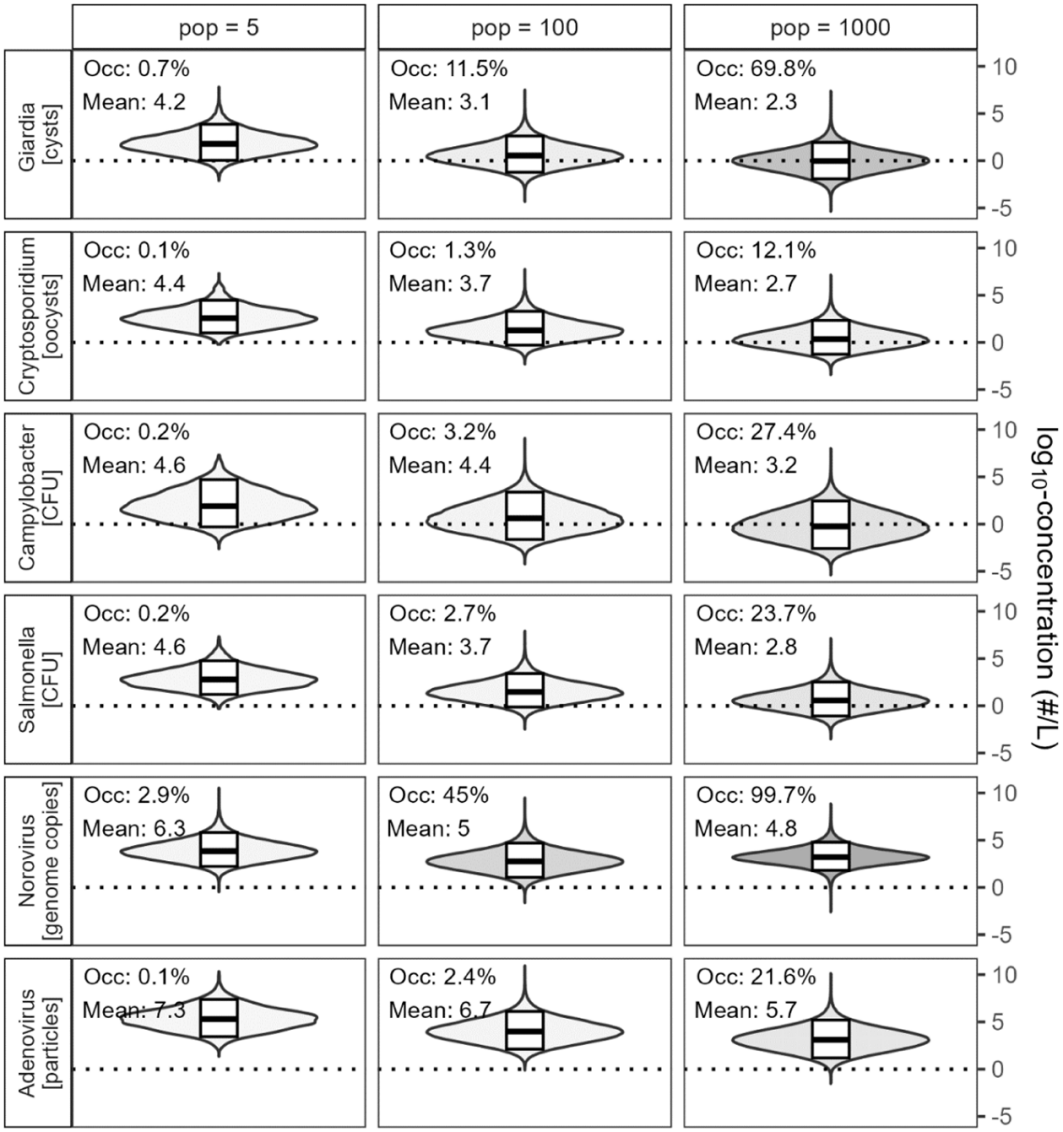
Distribution of pathogen concentrations when occurring in untreated greywater at three population sizes. Text annotations show pathogen occurrence (percentage of days per year with pathogens in the greywater) and log_10_-transformed arithmetic mean concentrations when occurring. Fill transparency is proportional to occurrence. Boxplots show 5%, 50% (median) and 95% quantiles of log_10_-transformed concentrations when occurring. CFU: colony-forming units; GC: genome copies. Epidemiology-based model adapted from Jahne et al.,^2^ with *E. coli* distributions from Sylvestre et al.^24^

### 2.2 Exposure Routes and Frequencies

The considered exposure routes were routine ingestion during nominal operation and during treatment failures. Exposure from accidental ingestion and cross-connections were not considered in this study, but could be incorporated if considered relevant for a specific context.

For baseline exposure to reclaimed greywater during nominal operation, we adopted the assumptions from Schoen et al.^11^ for routine indoor reuse, thus enabling comparison with previously published results: This scenario presumes that reclaimed water is reused for toilet flushing and clothes washing, with an ingestion volume *V_ing,baseline_* of 4 × 10^–5^ L/day and a frequency of use of 365 days/year.

To assess exposure during a failure, it is important to consider that users are only exposed to untreated or partially treated reclaimed water if they use the system while it is in failure. Thus, calculating exposure requires considering the probability that users access reclaimed water during a failure event, *P_use,failure_*, and the volume of water ingested during the system failure, *V_ing,failure_*. The probability that users use a system at least once during an undetected failure, i.e., *n_use,failure_* ≥ 1 was calculated as

2 $$p_{use,failure}=\{\begin{array}{ll} 0 & for\;t_{failure}=0\\ \frac{t_{failure}+t_{use}}{T_{Day}/n_{use}} & for\;t_{failure}+t_{use}<\frac{T_{Day}}{n_{use}}\\ 1 & for\;t_{failure}+t_{use}\geq \frac{T_{Day}}{n_{use}} \end{array}$$

where *t_failure_* and *t_use_* are the failure and use durations, respectively, *n_use_* and *n_use,failure_* are the number of uses per day and during an undetected failure, respectively, and *T_Day_* represent the reference period of 1 day. This model assumes that use events are spaced evenly throughout the day, hence the probability of users using the system at least once is 1 if the sum of the failure and use durations exceeds the use interval $\frac{T_{Day}}{n_{use}}$ , i.e., the two time intervals overlap.

If reclaimed water is used during a failure, users are exposed to an ingestion volume of *V_ing,failure_* untreated or partially treated water:

3 $$V_{ing,failure}=\frac{V_{ing,baseline}}{n_{use}}\times n_{use,failure}$$

where *n_use,failure_* was calculated as

4 $$n_{use,failure}=\lceil n_{use}\times \frac{t_{failure}}{T_{day}}\rceil$$

Note that eq 3 is conservative as it assumes that (1) any overlap between a failure and a use event results in full ingestion of the baseline volume during that event, and (2) the ingestion volume over multiple use events is ingested at once.

The use duration *t_use_* was assumed to be 60 s, with a frequency *n_use_* of 5 uses/day. A scenario analysis was conducted to evaluate the sensitivity of the results to these parameters, with *t_use_* set to 30 and 120 s, and *n_use_* to 2 uses/day and 10 uses/day. Published data on failures of on-site systems is limited, and reported frequencies depend on the specific context and treatment technology. This study therefore adopts a scenario-based approach with failure frequencies *n_failure_* of 1, 4, 12, and the boundary case of 365 failures per year that can inform technology developers on the level of robustness required for their systems to remain monitorable. All assumptions can be adapted to represent different use cases.

### 2.3 Treatment Trains and Failure Modes

Treatment trains consisted of one or two barriers with different failure modes followed by a reclaimed water storage tank. The storage tank was represented using a simplified buffering model with a hydraulic retention time in the reclaimed water storage tank, *HRT_storage_*, of 6 h, where dilution during a failure scales with the fraction of tank volume replaced (*t_failure_*/*HRT_storage_*). The residual LRV of a treatment barrier during a failure, *LRV_failure_* depends on the failure mode. We tested three model treatment trains for on-site greywater reuse to illustrate the effect of including several barriers with different types of failure modes, namely the failure of a bimodal treatment process ([Sec sec2.3.1]), chemical disinfection ([Sec sec2.3.2]), and a multibarrier treatment process ([Sec sec2.3.3]). These treatment train-specific assumptions can be adjusted for particular products or technologies.

For all failure modes, the ingested dose during a failure on day *i* was calculated as

5 $${dose}_{ing,failure,i}=\{\begin{array}{ll} V_{ing,failure}\times \frac{t_{failure}}{{HRT}_{storage}}\times 10^{\log_{10}\left(C_{P,GW,i}\right)-LRV_{failure}} & for\;t_{failure}<HRT_{storage}\\ V_{ing,failure}\times 10^{\log_{10}\left(C_{P,GW,i}\right)-LRV_{failure}} & for\;t_{failure}\geq HRT_{storage} \end{array}$$

where the ratio *t*_failure_/*HRT_storage_* accounts for the dilution of pathogens in the storage tank, and *C_P,GW,i_* are daily pathogen concentrations (see Section 2.1). Eq 5 assumes that, for failure durations longer than the hydraulic retention time, all water in the storage tank has been replaced, and therefore there is no remaining dilution effect. The dependency of the results on *HRT_storage_* was evaluated in a scenario analysis, where *HRT_storage_* was set to 0 or 12 h.

#### 2.3.1 One Barrier: Bimodal Treatment Process

The first treatment train consisted of a single bimodal unit process with complete failure, i.e., *LRV_failure_* in eq 5) was set to 0.

#### 2.3.2 One Barrier: Chemical Disinfection

The second treatment train consisted of chemical disinfection with a residual disinfectant. The assumption was that there is a chlorine contact zone with hydraulic residence time in the contact zone, *HRT_contact_*, of 10 min. The residual LRV time *t* after the onset of a failure was calculated based on the probability of passage of pathogens during a chemical disinfection failure, *π_f_* (*t*), as developed by Sylvestre et al.:^22^

6 $$\pi_{f}\left(t\right)={(\frac{1}{1+\frac{k\times C_{0}\times HRT_{contact}\times e^{-t/\left(HRT_{contact}/M\right)}}{M}})}^{M}$$

The first-order-kinetic rate constant *k* and the initial disinfectant concentration *C*_0_ were calculated to achieve the required LRT at time 0 (no failure), and *M*, a parameter to predict the hydraulics of the contact zone, was set to 6 to represent medium good hydraulics as proposed by Petterson and Stenström.^25^ A scenario analysis was conducted to evaluate the sensitivity of the results to these parameters, with *HRT_contact_* set to 5 and 30 min, and *M* to 2 (poor hydraulics) and 20 (near plug flow).

The arithmetic mean LRV during a failure of the chemical disinfection was numerically approximated by averaging a series of probabilities of passage computed at time steps of 1 s:

7 $$LRV_{failure,chem}=-\log_{10}\left(\overset{®}{\pi_{f}\;}\right)$$

The pathogen dose ingested during a failure was then calculated using eq 5 where *LRV_failure_* was replaced with *LRV_failure,chem_*.

#### 2.3.3 Two Barriers: Bimodal Process with Chemical Disinfection

The third treatment train included a bimodal treatment process that contributed a fixed LRV, *LRV_bimod_*, in nominal operation, combined with a chemical disinfection step contributing *LRV_chem_* log_10_-reductions. *LRV*_*chem*_ was calculated using eqs 6 and 7, where the parameters *k* and *C*_0_ were calculated to achieve *LRV_chem_* = *LRT* – *LRV_bimod_* at time *t* = 0. The pathogen doses ingested during treatment failures were calculated using eq 5 with *LRV_failure_* = *LRV_chem_* for a failure of the bimodal barrier, and *LRV_failure_* = *LRV_failure,chem_* + *LRV_bimod_* for a failure of the chemical disinfection.

### 2.4 Quantitative Microbial Risk Assessment Model

LRTs were calculated to achieve a tolerable annual risk of infection *P_inf_* of 10^–4^ infections pppy, where *P_inf_* represents the combined risk from baseline exposure (*P_inf,baseline_*) and from exposure to reclaimed water during failures (*P*_inf,failure_):

8 $$P_{inf}=1-\left(1-P_{inf,baseline}\right)\times \left(1-p_{use,failure}\times P_{inf,failure}\right)=10^{-4}\;\mathrm{pppy}$$

This equation represents a conservative simplification, as it assumes that the infection risk during failure is added to the baseline infection risk. SI 2 shows that this simplification has negligible effect on resulting LRTs (LRTs are increased by 0.1 or less).

#### 2.4.1 Baseline Probability of Infection

For baseline exposure, the annual probability of infection, i.e., over 365 days of baseline use per year, was calculated according to the standard equation developed by Schoen et al.:^11^

9 $$P_{inf,baseline}=S\times \left(1-\overset{365}{\underset{i=1}{\prod }}\left(1-DR\left(V_{ing,baseline}\times 10^{\log_{10}\left(C_{P,\mathrm{GW},i}\right)-LRT}\right)\right)\right)$$

with *S* the fraction of people in the exposed population susceptible to the reference pathogen (assumed = 1), and *DR*(...) the dose–response model for the reference pathogen. We used the following dose–response models: Rose et al.^26^ for *Giardia* spp., Messner and Berger^27^ for *Cryptosporidium* spp., Teunis et al.^28^ for *Campylobacter* spp., Haas et al.^29^ for *Salmonella* spp., Teunis et al.^30^ for norovirus, and Teunis et al.^31^ for adenovirus. Dose–response parameters are included in SI 1.

#### 2.4.2 Probability of Infection during Treatment Failures

For exposure to reclaimed water during treatment failures, the annual probability of infection was calculated according to the following equation:

10 $$P_{\inf,\mathrm{failure}}=S\times \left(1-\overset{n_{failure}}{\underset{i=1}{\prod }}\left(1-DR\left(dose_{ing,failure,i}\right)\right)\right)$$

In the case of two independent treatment barriers in series, consisting of a bimodal failure and a chemical disinfectant, the annual probability of infection for exposure during treatment failure was calculated individually for each failure using eq 10. The combined probability of infection was then calculated according to the following equation:

11 $$P_{inf,failure,comb}=1-\left(1-p_{use,failure,bimod}\times P_{inf,failure,bimod}\right)\times \left(1-p_{use,failure,chem}\times P_{inf,failure,chem}\right)$$

eq 8 was adapted to

12 $$P_{inf}=1-\left(1-P_{inf,baseline}\right)\times \left(1-P_{inf,failure,comb}\right)=10^{-4}\;\mathrm{pppy}$$

As for eq 8, this is a conservative simplification, as it assumes that the infection risk during failures is added to the baseline infection risk.

#### 2.4.3 Overview of the Full Quantitative Microbial Risk Assessment Model

Figure 2 summarizes how the input parameters and equations are combined in the overall QMRA model to link LRTs with failure durations (or monitoring frequencies). The QMRA model was implemented in R and is available at 10.25678/000GSK to reproduce all figures. LRTs were calculated by numerically solving eq 8 for each scenario (no failure, failure of a bimodal treatment barrier, failure of a chemical treatment barrier, failure of two barriers, with different failure frequencies, at three population sizes). To capture the variability of pathogen concentrations, LRTs were calculated using the full pathogen concentration data set from Section 2.1, i.e., simulating annual microbial risks over 10,000 years. 95% quantiles were computed empirically from the set of 10,000 LRTs per scenario. Baseline LRTs in the absence of treatment failures were calculated by setting the failure duration *t_failure_* to zero. Minimum required monitoring and response intervals correspond to the maximum failure duration for which the health benchmark can still be met 95% of the time. These intervals set the required frequency of operational monitoring to ensure each treatment barrier functions as intended.

**Figure 2:**
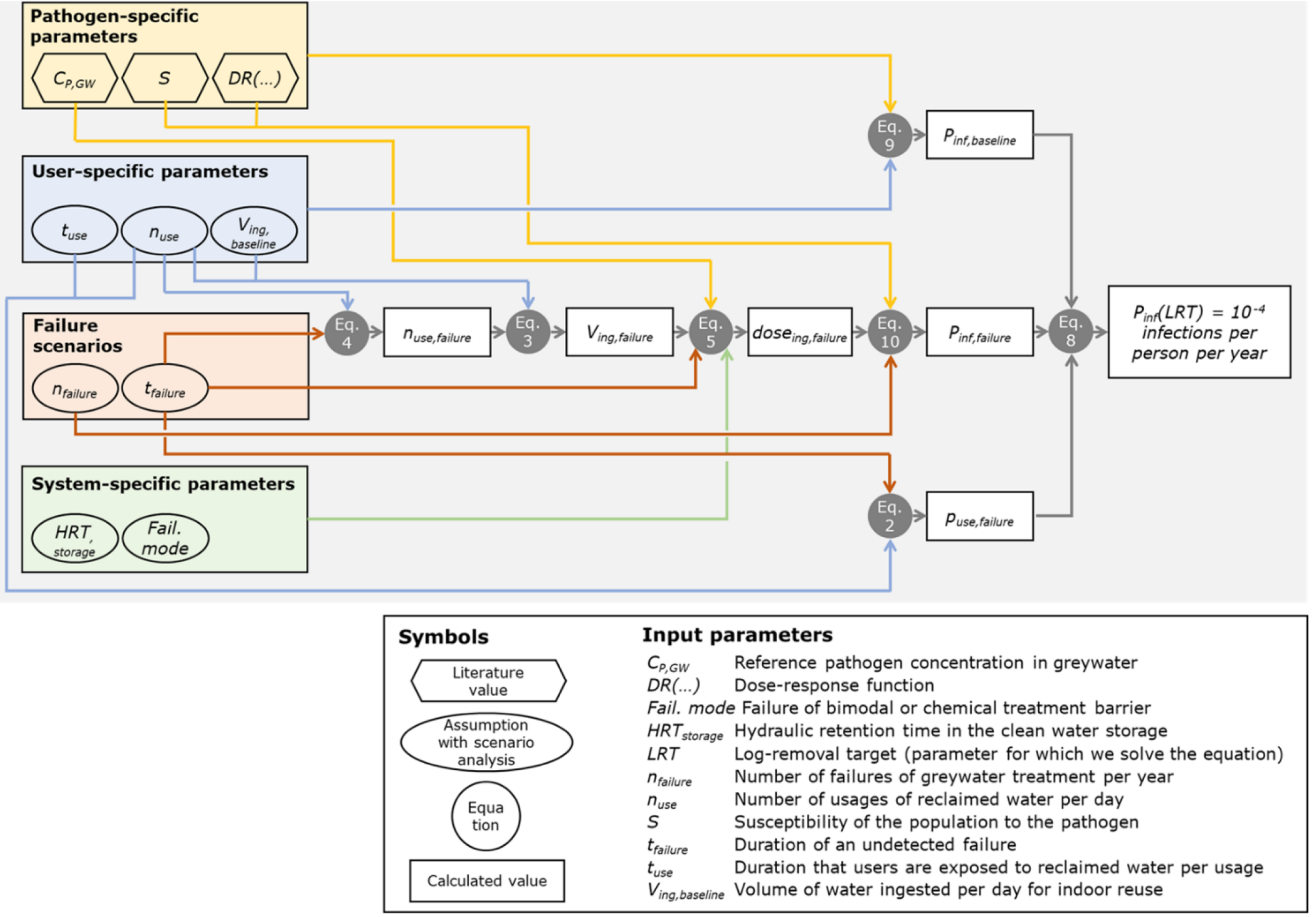
Overview of the QMRA model linking failure models with the required LRTs to meet a defined health benchmark. Model for a bimodal treatment barrier. Chemical treatment barriers additionally require eqs 6 and 7, and two-barrier systems require eqs 11 and 12.

To be consistent and allow comparison with previous studies on LRTs for greywater reuse,^3,7,9–11^ this study presents the 95% quantiles of LRTs. This approach is also consistent with existing U.S. guidance documents on on-site water reuse systems.^5,32^ If a treatment system maintains this minimum level of treatment during nominal operation, the predicted probabilities of infection across the population will be less than 10^–4^ pppy for each reference pathogen for 95% of the years.

## 3 Results and Discussion

Norovirus, the reference pathogen with the highest occurrence (Figure 1), required the highest monitoring frequencies across scenarios, making it critical for monitoring design. Accordingly, the main text focuses on norovirus, while results for other reference pathogens are provided in SI 3. Jahne et al.^33^ discuss the use of norovirus in QMRA, including its limitation. Norovirus has become a standard reference pathogen in water reuse QMRA since 2010 due to its prevalence in wastewater. While concerns exist that genome copy measurements may not accurately reflect infectious virus particles, meta-analyses of clinical trial and oyster outbreak data demonstrate that norovirus genomes from wastewater-impacted oysters remain highly infectious, supporting its use as a reference pathogen in QMRA. In this study, the modeled concentrations are based on genome copy measurements reported in fresh feces, further aligning with the inoculum used in dose–response challenge studies.

In the absence of treatment failures, the 95% quantiles of LRTs for norovirus were relatively similar across collection scales: 6.8 for 5 people, 6.9 for 100 people, and 7.2 for 1000 people (Figure 3, failure duration 0). This similarity reflects opposing scale-dependency on norovirus occurrence: smaller populations have lower pathogen occurrences (due to fewer total infections), but higher concentrations when occurring (due to less dilution by the noninfected part of the population) (Figure 1). For the three considered population sizes, this results in similar LRTs in spite of widely different occurrences (2.9% to 99.7%) and mean concentrations (6.3 log_10_ GC/L to 4.8 log_10_GC/L). Note that the baseline LRTs are lower than those reported in Reynaert et al.,^7^ as the previous study included uses of reclaimed water with higher volumes of routine ingestion.

**Figure 3:**
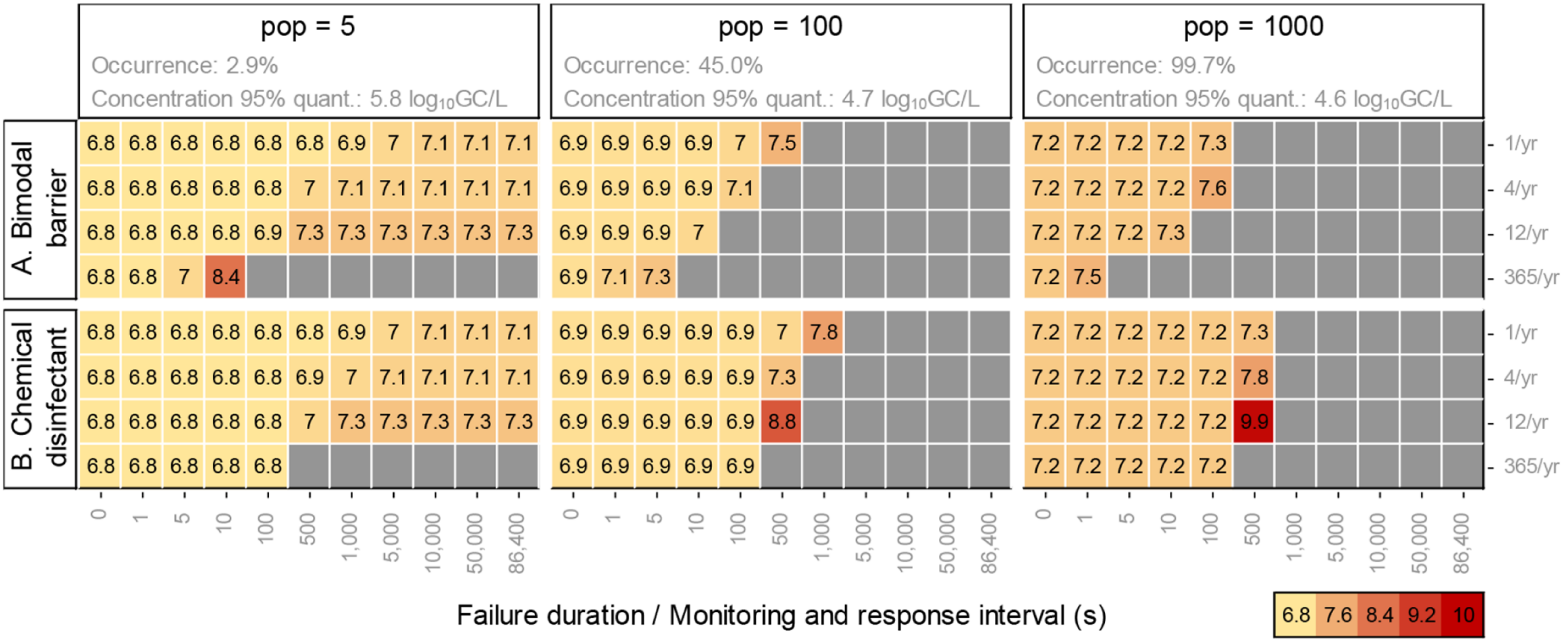
95% quantiles of norovirus LRTs for the recycling of greywater at three collection scales for the complete failure of (A) a bimodal barrier or (B) a chemical disinfectant barrier for different failure durations (equivalent to the monitoring and response interval) (*x*-axes) and failure frequencies (*y*-axes). LRTs for failure durations of 0 correspond to baseline LRTs in the absence of treatment failures. GC: genome copies. Gray boxes indicate that the health benchmark cannot be met for the respective failure duration (A) or that the required LRT is equal to or larger than 10 (B).

### 3.1 Results for One Bimodal Treatment Barrier

While LRTs for norovirus were similar across scales during nondisrupted operation, understanding how the lower occurrence of pathogens influences LRTs that account for the risk from treatment failures is important. Figure 3A presents the required LRTs as a function of failure duration (representing the monitoring and response interval) for bimodal treatment failures, allowing to quantify the trade-offs between more frequent monitoring vs higher treatment requirements (LRTs for all reference pathogens in SI 3.2). Gray boxes indicate scenarios where the health benchmark of 10^–4^ infections pppy cannot be met for the corresponding failure duration because the probability of infection from the failure alone exceeded the health benchmark. The minimum required monitoring and response interval is defined as the maximum failure duration for which the health benchmark can still be met.

Bimodal treatment failures had different effects depending on the scale of reuse. At all scales, LRTs increased with longer failure duration, as both the probability of use during a failure and exposure volumes increase. The required LRTs increased more strongly as a function of failure duration for larger population sizes because norovirus occurrence increases with scale. At a scale of 5 people, norovirus is absent most of the time (Figure 1), so treatment failures have a lower impact human health.

For the 100- and 1000-people scales, indoor reuse of greywater required high monitoring frequencies even when assuming only one failure per year: for the 100-people scale, the required monitoring interval was <1000 s, while it was <500 s for the 1000-people scale. Higher failure frequencies were associated with shorter required monitoring intervals. For instance, for 12 failures per year, the required intervals were <100 s at the 100- and 1000-people scales. In contrast, the low occurrence of norovirus at the scale of 5 people led to longer monitoring intervals with moderate increases in LRTs. For instance, increasing the LRT by 0.3-log_10_ units (from 6.8 to 7.1) for norovirus allowed to increase the required monitoring interval to >1 day for systems that fail once per year, while an increase of 0.5-log_10_ units (from 6.8 to 7.3) was needed to increase the monitoring interval to >1 day for 12 failures per year.

The results indicate that norovirus determines the minimum monitoring frequency in most scenarios, due to its relatively high prevalence. Except for adenovirus, all other pathogens were associated with lower LRTs and, consequently, lower monitoring requirements (SI 3). Adenovirus—the pathogen requiring the highest LRTs in the absence of failures in the larger-scale systems—only required higher monitoring frequencies at the extreme (but unrealistic) failure frequency of 365 failures/year. This suggests that future work to improve risk assessment of on-site reuse systems including treatment failures should focus on better characterization of norovirus risks.

Overall, the operational monitoring frequencies for systems relying on a single bimodal treatment barrier are impractically high when considering the limitations of current monitoring technologies, such as realistic sensor response times (see Section 3.5.2). Under these conditions, single bimodal barriers are unsuitable for viruses with existing monitoring approaches, although they can still be a manageable option for controlling bacterial and protozoan risks in on-site systems (SI 3). Results for a more realistic treatment train consisting of two barriers are presented in Section 3.3.

### 3.2 Results for One Barrier with Chemical Disinfectant

Results differed between chemical disinfectant barriers and bimodal barriers (Figure 3B; results for all reference pathogens in SI 3.3). Unlike the bimodal barrier, the health benchmark could theoretically always be met with a sufficiently high initial disinfectant dose such that a residual remains throughout the failure period. To generate reasonable results for the visualization we capped the LRTs at 10, although this is still higher than the virus LRVs that can realistically be achieved with chemical disinfectants. The selection of treatment trains must consider the efficacy of the selected treatment technologies in achieving the required LRTs. For this reason, many regulatory agencies cap log-removal credits, for instance at 4 ^34^ or at 6 ^35^ LRVs to minimize risks from treatment failures.

Due to the presence of a disinfectant residual that buffers short-term treatment failures, the required monitoring intervals were longer than for bimodal treatment barriers, especially at higher failure frequencies. For the 5-people scale, increasing the norovirus LRT by 0.3-log_10_ units (from 6.8 to 7.1) allowed to increase the monitoring interval to >1 day for up to 4 failures per year, while an increase of the LRT by 0.5-log_10_ units was needed in the case of 12 failures per year. In the larger-scale systems, the monitoring interval for 12 failures per year was <500 s for a chemical disinfectant compared to <100 s for a bimodal barrier at the 1000-people scale. While the monitoring intervals for chemical disinfectants are only moderately longer than those for bimodal treatment barriers, these differences may nonetheless be relevant for the monitorability of on-site reuse systems, considering realistic sensor response times (see Section 3.5.2).

### 3.3 Results for Two Barriers Consisting of One Bimodal Process with Chemical Disinfection

To represent more realistic greywater treatment trains, we investigated the effect of incorporating two barriers on the treatment and monitoring requirements. Such multibarrier approaches are often prescribed in centralized water reuse schemes. In this configuration, a bimodal barrier contributes 3 LRVs of norovirus removal, while chemical disinfection provides the remaining required LRVs (Figure 4). This treatment train could for instance represent a membrane bioreactor combined with chlorination. MBRs with chlorination represent a realistic option for on-site greywater treatment across all covered scales. MBRs have been identified as an effective solution for building-scale greywater reuse,^36^ with applications ranging from single-family homes^37,38^ to several hundred users.^39^ MBRs are often combined with disinfection such as chlorination or UV to meet microbial water quality targets.^36^ Results with other allocations of LRVs across treatment barriers (2 or 4 LRVs for the bimodal treatment barrier) are presented in SI 4.

The monitoring requirements for norovirus are considerably lower compared to the single-barrier scenarios. For a household-scale system, daily monitoring of both treatment barriers is sufficient with a maximum increase of only 0.5 log_10_-units for up to 12 failures per year. Similarly, daily monitoring of the bimodal treatment barrier is feasible for 100-people systems, if the chemical disinfectant barrier is monitored every 100 s (up to 12 failures/year). If the bimodal barrier contributes to more LRVs, the required monitoring for that barrier increases, while the monitoring requirements for the chemical disinfectant decreases, and vice versa (see SI 4). Overall, Figure 4 illustrates the option space between treatment barriers, LRTs and failure durations that all ensure meeting the health benchmark.

**Figure 4:**
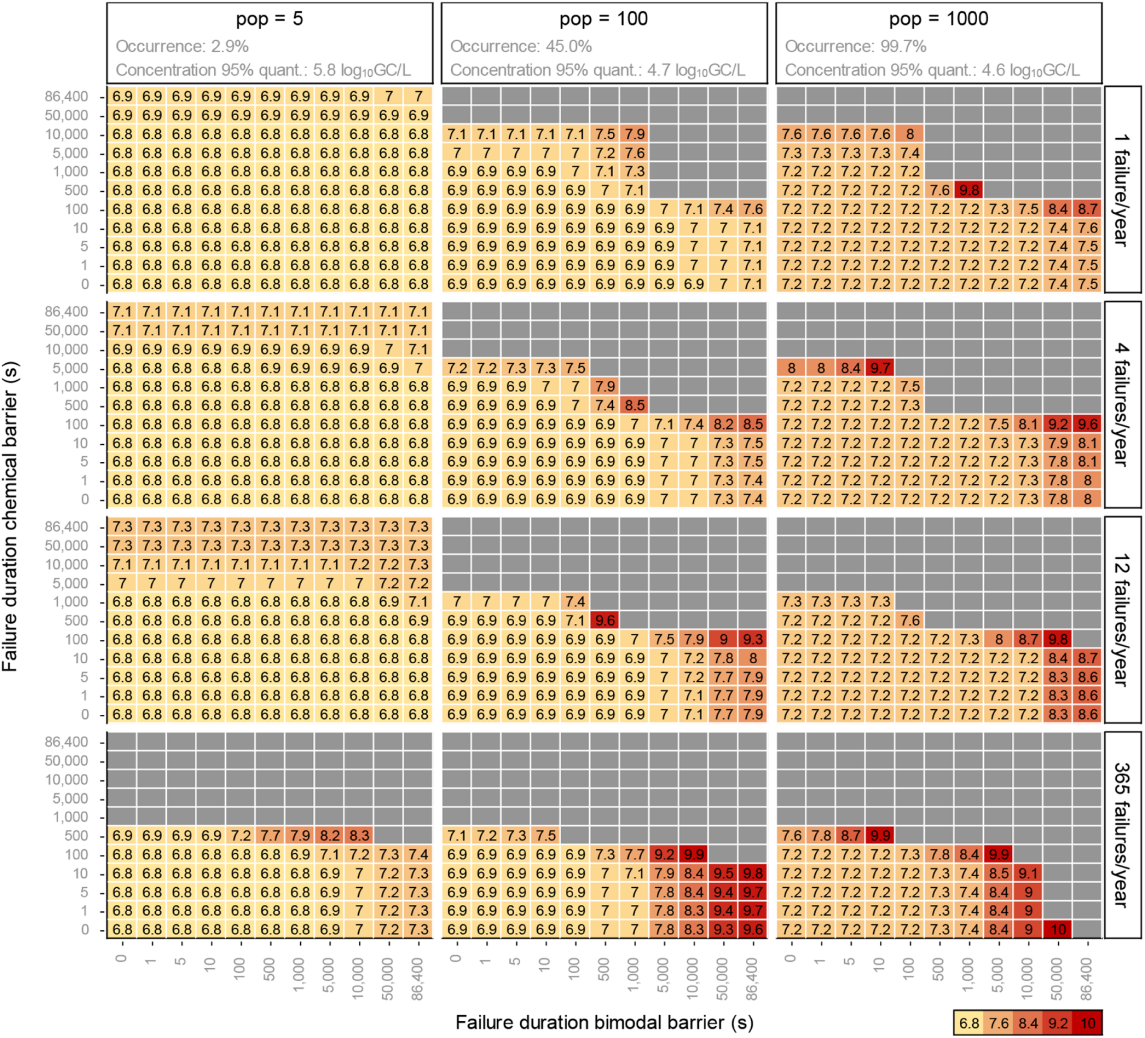
95% quantiles of norovirus LRTs for the recycling of greywater at three collection scales for the complete failure of a bimodal failure (contributing 3 LRVs) and a barrier with residual disinfectant (contributing the remaining required LRVs). GC: genome copies. Gray boxes indicate that the required LRT is equal to or larger than 10.

### 3.4 Sensitivity Analysis

Uncertainty in LRT estimates stems from several factors. The sensitivities of the epidemiology-based and QMRA models applied here are discussed in detail in Jahne et al.^2^ and Reynaert et al.,^7^ with major sources of uncertainty including exposure volumes and routes, *E. coli* concentrations in greywater sources, pathogen shedding rates, and dose–response models, particularly at low pathogen doses. Accordingly, the sensitivity analysis focused on parameters specific to this study. The modeling outcomes were influenced by various assumptions, including user-specific (use duration and frequency), system-specific (hydraulic retention time in the storage tank), and treatment-specific (hydraulic retention time and flow conditions in the chlorine contact zone) factors. The sensitivity analysis showed that system- and treatment-specific assumptions had a greater impact on the results than user-specific assumptions (Table 1; full results in SI 5).

**Table 1:** Sensitivity of Minimum Monitoring and Remediation Intervals to Model Input Parameters

Parameter	Scenario low	Main scenario	Scenario high	Effect^b^,^a^	Explanation
**User-specific**					
*t*_*use*_	30 s	60 s	120 s	–	Longer and/or more frequent use increases the probability that reclaimed water is used during failure.
*n*_*use*_	2 x/day	5 x/day	10 x/day	–	
**System-specific**					
*HRT*_*storage*_	0 h	6 h	12 h	++	A longer storage HRT results in greater dilution of the partially/untreated water.
**Treatment-specific**					
*HRT*_*contact*_	5 min	10 min	30 min	++	A longer contact HRT leads to a slower decline in reclaimed water quality.
*M*	2	6	20	––	Better hydraulics result in less mixing (i.e., dilution) in the contact zone.

a Effect: +/++: Indicate an increase in required monitoring interval with increasing parameter value; −/––: Indicate a decrease. Full results for 95% LRTs are presented in SI 5.

b +/–: Monitoring requirements only change in the 365 failures/year scenarios. ++/––: Monitoring requirements also change in the <365 failures/year scenarios.

At the tested temporal resolutions, the required monitoring intervals did generally not substantially change as a function of the investigated parameters. Exceptions were the HRTs of the storage and contact tanks, where longer HRTs increased buffering, leading to slower changes in water quality and therefore lower required monitoring, as well as the hydraulics of the disinfection unit. In contrast when the storage tank provided no buffering (i.e., *HRT_storage_* of 0) even 1-s failures caused exceedances of the risk benchmark for the 100- and 1000-people systems. Ensuring a minimum hydraulic retention time in the storage tank is therefore critical. Because real storage tanks have residence-time distributions and short-circuiting (residence times shorter than the mean HRT), the effective buffering may be lower than represented here as the main scenario, potentially leading to more stringent monitoring frequencies. This underscores the importance of engineered storage buffers—such as the water storage tank modeled in this study—not only for smoothing variations between treatment and demand, but also as an important component of risk management, with trade-offs between tank size/design and required LRTs/monitoring frequencies.

An aspect not covered in this sensitivity analysis is the impact of process dependencies in multibarrier treatment. For example, the efficacy of chlorination would likely be affected by a failure of the membrane bioreactor. Such dependencies could be incorporated by correlating process LRVs,^40^ as done for a direct potable reuse treatment train by Zhiteneva et al.^41^

### 3.5 Practical Implications for the Design of On-Site Greywater Reuse Systems

This study explores the effect of treatment failures on required LRTs for onsite greywater reuse considering realistic pathogen variability and use patterns. The QMRA model can inform the design of greywater reuse systems by quantifying the trade-offs between a range of design choices, including system LRVs, monitoring frequency, scale, and technology selection (Section 3.5.1). However, the practical feasibility of implementing the proposed approaches must also be considered (Section 3.5.2).

#### 3.5.1 Trade-Offs in Designing Greywater Reuse Systems

Current greywater reuse systems vary widely in scale and technology. In terms of scale, systems can serve from as few as two people sharing a household-scale system to several hundred users in building- or neighborhood-scale systems.^42^ In terms of technology, greywater treatment systems typically combine physical and biological processes to remove solids, biodegradable organic carbon and nutrients, followed by disinfection to inactivate pathogens.^43^ The present study enables the quantification of trade-offs around the monitoring of greywater reuse systems, highlighting three key aspects. It is important to note that these trade-offs address only risks from enteric pathogens; opportunistic pathogens, such as *Legionella pneumophila*, are beyond the scope of the proposed framework because their control relies on building-system management (e.g., temperature, hydraulics) rather than LRTs.^5,32^

First, this study quantifies the trade-offs between increasing monitoring frequency and incorporating additional treatment required beyond the LRTs necessary to meet the health benchmark under nominal operation. The model results demonstrate that it is possible to increase the required monitoring frequency by increasing nominal LRVs. For instance, at the scale of 5 people, monitoring intervals can be increased to >1 day for most scenarios, with only minor LRV increases. Given the small magnitude of these increases, short treatment disruption are likely already buffered by typical safety margins in operational bounds,^21^ or by rounding LRTs when translating QMRA results into practical guidance (e.g., rounding to next 0.5-log, as suggested by Sharvelle et al.^32^). At larger scales, extending monitoring intervals by increasing nominal LRVs is more limited, but can still improve monitorability, e.g., by enabling monitoring intervals of 100 or 500 s with only moderate increases in LRVs.

Second, this study quantifies the trade-offs between more frequent monitoring and additional treatment barriers, and enables comparison of monitoring frequencies for different treatment barriers. Adding a residual disinfectant or implementing multiple barriers can reduce required monitoring intervals with minimal additional treatment, thereby increasing the monitorability of on-site greywater reuse. This underscores the importance of multibarrier systems, as commonly required for centralized water reuse schemes, to reliably meet health benchmarks with implementable monitoring requirements. Monitoring frequencies could be further reduced by incorporating treatment redundancy, as is standard in centralized reuse schemes.

Third, the study enables a comparison of LRTs and monitoring requirements between small and larger greywater collection systems. While LRTs are similar across scales for viruses in the absence of failures, the results show that household-scale systems require less frequent monitoring due to the lower occurrence of viruses. This finding is promising for small-scale systems, where online monitoring at high frequencies can be particularly challenging (see Section 3.5.2). Here, additional treatment provides greater flexibility for system producers and operators in choosing appropriate monitoring solutions, particularly for small systems such as single-family home systems, where real-time monitoring may be unfeasible or cost-prohibitive. Advantages for household-scale systems may be further increased if less conservative risk benchmarks are applied, given the reduced relative importance of reclaimed water in pathogen transmission compared with other routes (e.g., person-to-person, fomites).^44^ However, the results also emphasize that online monitoring is necessary at larger scales; at a 1000-people scale, even a single <10 min treatment failure would exceed the selected annual risk benchmark if there is only one pathogen barrier. Although these reduced monitoring frequencies offer practical advantages for household-scale systems, they are unlikely to be a decisive factor in determining the most appropriate scale of greywater reuse. Garrido-Baserba et al.^45^ compared the costs for decentralized systems for rainwater harvesting, and grey- and blackwater treatment at scales ranging from 2.3 to 300 users. Their results indicate that capital expenditure per person of the smallest compared to the largest scales was 10 times higher, while the operating costs were even 30 times higher. While fully autonomous monitoring can contribute to reducing operation and maintenance cost, it will likely not significantly change the overall cost differences between household-scale and building-scale systems. Ultimately, the optimal trade-offs depend on the business model of the technology provider and operator, provided health benchmarks are reliably met. Household-scale systems offer the greatest flexibility, as minor operational changes to increase nominal LRVs (e.g., by prolonging contact time in UV disinfection or increasing chlorine dose) can allow for substantially longer monitoring and remediation intervals, thereby facilitating implementation of monitoring for such systems (see Section 3.5.2).

#### 3.5.2 Feasibility of Implementation

The determination of additional LRTs beyond those required for nominal operation was based on three principles (Section 2): (1) that including additional log-removal capacities within the same unit treatment is feasible, (2) that sensors capable of reliably detecting failures of the treatment barriers at the tested monitoring frequency exist, and (3) that the use of reclaimed water is immediately halted upon detection of a failure.

The first principle– that additional log-capacities are incorporated within the same treatment unit—is different from standard practice in centralized potable reuse systems, which typically include process redundancy in the form of independent treatment barriers.^46^ For instance, Pecson et al.^23^ showed that approximately 4-log_10_ units of independent treatment redundancy was required to meet an annual health benchmark of 10^–4^ infections pppy accounting for a 15 min 6-log_10_ removal failure.

For on-site systems, the inclusion of independent treatment redundancy is likely not feasible due to the lack of economies of scale. However, this study shows that in on-site nonpotable systems, where pathogen occurrence is lower than in centralized systems and exposure to reclaimed water is lower than in potable systems, the impact of treatment failures can be buffered by including additional treatment capacity within the same unit treatment. While this approach is effective, it is important to recognize that it is not feasible to easily add log_10_-removal capacities for all types of treatment barriers. For membrane processes, for instance, pathogen LRVs cannot be adjusted only through operational changes. Instead, additional LRVs could be incorporated by using membranes with smaller pore sizes, alternative materials or modified membrane surface.^47^

In contrast, flexibly adjusting the LRVs is possible for many conventional disinfection technologies, including chlorination, UV, and ozonation, within certain limits. It should be noted, however, that many of the LRTs reported herein exceed the removal credits achievable by a single unit process and multiple pathogen barriers may nonetheless be required.

The validity of the second principle—that suitable sensors for monitoring exist—depends on the type of treatment barrier. Not all types of treatment barriers can be effectively monitored online. For example, the WHO guidelines for potable reuse give membrane bioreactors only 1.5 LRV credits for virus removal, rather than the 5 LRVs from challenge testing, because online monitoring with turbidity or transmembrane pressure is not sensitive enough for higher credits.^48^ In contrast, established online sensors are available for the monitoring of several common disinfection technologies, including chlorination, UV, and ozonation.^48^ When evaluating the suitability of sensors to monitor water treatment barriers, sensor response times are an important factor. This study shows that required monitoring frequencies can be <100 s (e.g., bimodal barrier, 100-people scale, 12 failures/year), which can be limiting for many sensors. For instance, oxidation–reduction potential sensors, which are an attractive alternative to chlorine sensors due to lower costs, can have response times of 10 min or more,^49^ which may be too slow for some of the scenarios modeled in this study. This problem—that some treatment technologies cannot be adequately monitored at low cost, with low maintenance, and at sufficient frequency—highlights the importance of integrated design and monitoring, in which monitoring is considered as an integral part of treatment train design rather than added retrospectively.

The implementation of online sensors in on-site systems poses challenges compared to centralized systems. One challenge is the requirement for regular maintenance to maintain accuracy.^50^ However, several solutions can be implemented for increased robustness in the absence of operators, including monitoring redundancy, plausibility checks by anomaly detection, or the introduction of deliberate system dynamics to test that sensors are reacting as expected.^51^ Another challenge is the high cost of many online sensors. Here, emerging technologies, such as low-cost chlorine sensors optimized for on-site reuse systems, offer potential alternatives.^52^

Overall, the second assumption is not always valid. It is therefore important to consider the ability to monitor water treatment barriers at suitable sensitivity, frequency, accuracy, and cost when selecting technologies for on-site greywater reuse.

The third principle—that water supply is immediately interrupted once a failure has been detected—can be discussed from two perspectives. From the user perspective, it is critical that water is available at all times. This can easily be ensured, as the greywater reuse systems only provides water for nonpotable uses such as toilet flushing. There remains a need for a drinking water supply that provides water for direct consumption, bathing, and other direct contact uses. Depending on the setup, there may be dual water plumbing, or a direct connection to the reclaimed water storage tank, where water is automatically discharged to the sewer when a failure is detected and replaced with drinking water. From the system operator’s perspective, automatic service interruption requires on-site operator intervention to restart the system after a failure. Therefore, only major treatment failures, such as those modeled herein, should result in insufficient water quality. This highlights the importance of overall system robustness. However, as for other monitoring-related trade-offs (see Section 3.5.1), the ultimate selection of suitable management strategies depends on the business model of the technology provider and operator. As an alternative to interrupting the water provision, system operators could also include their own response time in the monitoring and response interval, increase the monitoring frequency to allow for intervention time, or implement automated corrective actions, if this is feasible and economically attractive.

## 4 Conclusions

- This study presents a risk-based framework for determining LRTs as a function of monitoring frequency in on-site water reuse systems based on a defined treatment design and failure management approach. The framework is exemplified using greywater reuse for indoor nonpotable applications; however, it can also be applied to other configurations.
- Addressing treatment failures in on-site water reuse systems is challenging due to the lack of personnel for routine monitoring. However, this study demonstrates that small collection scales also present advantages in terms of required monitoring frequencies, due to the low occurrence of enteric pathogens in such systems, decreasing the impact of treatment failures on annual infection risks.
- System robustness, treatment trains incorporating multiple barriers, and engineered storage buffers are key to making on-site water reuse systems monitorable.
- The risk-based framework can inform (i) developers and operators of on-site reuse systems in selecting appropriate treatment trains and designing operation and monitoring schemes based on acceptable trade-offs between treatment and monitoring, and (ii) regulators in specifying LRTs as a function of the monitoring frequency.

## Supplementary Material



## Notes

Epidemiology-based models and dose–response models; LRTs for all reference pathogens; alternative scenarios for the two-barrier system; sensitivity analysis (PDF)

## #

Eawag, Swiss Federal Institute of Aquatic Science and Technology, 8600 Dübendorf, Switzerland

## ∇

Office of Chemical Safety and Pollution Prevention, U.S. Environmental Protection Agency, 26 W. Martin Luther King Drive, Cincinnati, OH 45268, USA

## List of symbols

*C*_*EC,F*_: *E. coli* density in feces
*C*_*EC,GW*_: *E. coli* concentration in greywater
*C*_*P,F*_: Pathogen density in feces
*C*_*P,GW*_: Pathogen concentration in greywater (*C*_*P,GW,i –*_ *on day (i)*
*dose*_*ing,failure,i*_: Ingested pathogen dose during a treatment failure on day *i*
*DR­(...)*: Dose–response model
*HRT*_*contact*_: Hydraulic retention time in the chemical disinfection contact zone
*HRT*_*storage*_: Hydraulic retention time in the reclaimed water storage tank
*k, C*_0_: First-order-kinetic rate constant and initial disinfectant concentration to achieve the required log-removal target at time 0
*LRT*: Log-removal target
*LRV*: Log-removal value (*LRV*_*bimod*_–—of a bimodal barrier; *LRV*_*chem*_—of a chemical disinfectant)
*LRV*_*failure*_: Residual log-removal value during a failure (*LRV*_*failure,chem*_—failure of a chemical disinfectant)
*M*: Hydraulics of the chemical disinfection contact zone
*N*: Total daily infections of a reference pathogen in a selected population size
*n*_*use*_: Number of uses of reclaimed water per day (*n*_*use,failure*_—while the system is in failure)
*P*_*inf*_: Annual probability of infection
*P*_*inf, baseline*_: Annual probability of infection from baseline exposure
*P*_*inf,failure*_: Annual probability of infection from exposure during treatment failures (*P*_*inf,failure,bimod*_—failure of a bimodal barrier; *p*_*use,failure,chem*_—failure of a chemical disinfectant; *P*_*inf,failure,comb*_—failure of two combined barriers)
*P_use,failure_*: Probability of at least one use during an undetected failure (*p_use,failure,bimod_* —failure of a bimodal barrier; *p_use,failure,chem_* —failure of a chemical disinfectant)
*S*: Susceptibility of the population to a reference pathogen
*T*_*day*_: Reference period of 1 day
*t*_*failure*_: Failure duration (equivalent to detection and remediation time)
*t*_*use*_: Duration of each instance of reclaimed water use
*V*_*ing,baseline*_: Baseline ingestion volume of reclaimed water
*V*_*ing,failure*_: Ingestion volume of untreated or partially treated water during a failure
π_*f*_: Probability of pathogen passage during the failure of a chemical disinfectant barrier
