# Supplementary material for: Monitoring Frequencies for On-Site Water Reuse: A Risk-Based Framework Applied to Greywater Reuse
Source: ACS ES T Water. 2026 Apr 22;6(5):3031–43. doi: 10.1021/acsestwater.5c01511 (PMC13162339; doi:10.1021/acsestwater.5c01511)
Supplement: Supplementary file 1 [file ew5c01511_si_001.pdf]

Supplementary Information

Monitoring frequencies for on-site water reuse: a risk-based framework applied to greywater reuse

Eva Reynaert[a,b,c,*], Michael A. Jahne[d,e], Émile Sylvestre[f,g]

[a] Technische Universität Berlin, Water Treatment, 10623 Berlin, Germany

[b] German Environment Agency, Section II 3.3 (Water treatment), 12307 Berlin, Germany

[c] Eawag, Swiss Federal Institute of Aquatic Science and Technology, 8600 Dübendorf, Switzerland (current address)

[d] Office of Research and Development, U.S. Environmental Protection Agency, 26 W. Martin Luther King Drive, Cincinnati, OH 45268, USA

[e] Office of Chemical Safety and Pollution Prevention, U.S. Environmental Protection Agency, 26 W. Martin Luther King Drive, Cincinnati, OH 45268, USA (current address)

[f] Delft University of Technology, Sanitary Engineering, 2628 CN Delft, The Netherlands

[g] KWR Water Research Institute, 3433 PE Nieuwegein, The Netherlands

[*] Corresponding author: eva.reynaert@eawag.ch

The supplementary information (SI) contains five sections:

- SI 1: Epidemiology-based model and dose-response models
- SI 2: Numeric effect of model simplification on LRTs
- SI 3: LRT results for all reference pathogens
- SI 4: Different allocations of LRVs between a bimodal barrier and a chemical disinfectant
- SI 5: Sensitivity analysis

SI 1: Epidemiology-based model and dose-response models

Due to the limited availability of measured pathogen concentrations for individual greywater streams, especially at small scales, we simulated pathogen concentrations following an epidemiology-based approach developed by Jahne et al.[1] with *E. coli* distributions from Sylvestre et al.[2]. In short, the epidemiology-based model consists of three steps:

1) Simulating total daily infections N of each reference pathogen in a selected population size over 10,000 years based on generalized incidence rates and infection durations reported in the literature.
2) Simulating the daily mass concentration of feces in greywater over 10,000 years based on the distribution of measured *E. coli* concentrations in greywater, $C_{EC,GW}$, relative to the density of *E. coli* in feces, $C_{EC,F}$.
3) Inferring pathogen contributions to greywater by summing up fecal contributions from the infected part of the population and combining with the densities of pathogens shed in feces.

These steps result in the final equation for pathogen concentrations in greywater:

$$C_{P,GW} = \left(\frac{1}{pop}\right) \sum_{i=1}^{N} \frac{C_{P,F,i} C_{EC,GW,i}}{C_{EC,F,i}} \qquad (1)$$

Table 1 presents the modelled *E. coli* concentrations in the considered greywater sources ($C_{EC,GW}$) along with the relative contributions of individual sources to combined greywater.

Table 1. Modeled *E. coli* concentrations in individual greywater and relative contribution to combined greywater.

Greywater source and reuse application	Contribution to combined greywater [a]	Lognormal distribution (with a PERT distribution of μ for BS) μ_{min}	mode μ	μ_{max}	σ	Source
Washing machine	29%		0.04		4.9	Sylvestre et al.[2]
Bathroom sink	33%	1.68	5.18	7.54	1.12	Jahne et al.[1]
Shower	38%		4.49		3.22	Sylvestre et al.[2]

[a] Based on relative contributions from Jahne et al.[1]

The incidence, duration of excretion and the density of the reference pathogens in feces are presented Table 2 along with the pathogen dose-response models.

Table 2. Incidence, duration and pathogen density in feces used for pathogen concentration simulation, along with dose-response model of each reference pathogen. NORM: normal distribution with mean, sd. TRIANG: triangular distribution with min, mode, max. PERT: PERT distribution with min, mode, max. CFU: colony-forming units.

**Reference pathogen**	**Pathogen concentration simulation (distributions from Jahne et al.[1])**			**Susceptibility**	**Dose-response model[a]**	
	**Incidence**	**Duration**	**Density in feces**	**S**	**Model**	**Parameters**
**Protozoa**						
Giardia spp.	PERT(27, 38, 50)<br>$10^4 p^{-1} y^{-1}$<br>Scallan et al.[3]	NORM(4.5, 0.7)<br>ln days<br>Schönning et al.[4]	NORM(15, 1.7)<br>ln cysts wet-g^{-1}<br>Schönning et al.[4]	1	exponential	r = 0.0199<br>Rose et al.[5]
Cryptosporidium spp.	PERT(5, 23, 65)<br>$10^4 p^{-1} y^{-1}$<br>Scallan et al.[3]	TRIANG(5, 10, 30)<br>days<br>Extreme duration:<br>60 days for 2% of cases<br>Petterson et al.[6]	TRIANG(6, 7, 9)<br>log_{10}-oocysts wet-g^{-1}<br>Petterson et al.[6]	1	fractional Poisson	P = 0.737<br>U = 1<br>Messner and Berger[7]
**Bacteria**						
Campylobacter spp.	PERT(14, 35, 68)<br>$10^4 p^{-1} y^{-1}$<br>Scallan et al.[3]	TRIANG(15, 34, 42)<br>days<br>Extreme duration:<br>60 days for proportion of cases<br>TRIANG(0.005, 0.0075, 0.01) days<br>Petterson et al.[6])	TRIANG(4, 6, 10)<br>log_{10}CFU wet-g^{-1}<br>Petterson et al.[6]	1	hypergeometric	α = 0.38<br>β = 0.51<br>Teunis et al.[8b]
Salmonella spp.	PERT(23, 37, 60)<br>$10^4 p^{-1} y^{-1}$<br>Scallan et al.[3]	TRIANG(10, 15, 50)<br>days<br>Petterson et al.[6]	TRIANG(6, 7.5, 9)<br>log_{10}CFU wet-g^{-1}<br>Petterson et al.[6]	1	approximate beta-Poisson	α = 0.3126<br>β = 2884<br>Haas et al.[9]
**Viruses**						
Adenoviruses	PERT(41, 97, 210)<br>$10^4 p^{-1} y^{-1}$<br>Hall et al.[10]	TRIANG(3, 7, 12)<br>days<br>Petterson et al.[6]	TRIANG(8, 10, 12)<br>log_{10}particles wet-g^{-1}<br>Petterson et al.[6]	1	hypergeometric	α = 5.11<br>β = 2.80<br>Teunis et al.[11 c]
Norovirus[d]	PERT(428, 696, 1025)<br>$10^4 p^{-1} y^{-1}$<br>Scallan et al.[3]	PERT(13, 28, 56)<br>days<br>Atmar et al.[12]	PERT(7.5, 9.75, 12)<br>log_{10}genome copies wet-g^{-1}<br>Atmar et al.[12]<br>Extended density after first two weeks:<br>PERT(3.5, 6.5, 7.6)<br>log_{10}genome copies wet-g^{-1}<br>Atmar et al.[12]	1	hypergeometric	α = 0.393<br>β = 0.767<br>Teunis et al.[13 d]

[a] Refer to Schoen et al.[14] for a discussion of the uncertainties around the use of genome-copies for the epidemiology-based model and dose-response model of norovirus.

[b] Parameters for the outbreak test data, as this data is more conservative than the challenge test data.

[c] Note that the generalized dose-response model from Teunis et al.[11] combines information from inhalation, oral ingestion and droplet inoculation. Due to the limited data for oral ingestion, the model parameters are primarily driven by the inhalation route. We have adopted this model because it currently represents the best available information, however, the model may need to be updated once dose-response models specific to oral ingestion of adenoviruses become available.

[d] For the norovirus dose-response model, we followed recommendations from Schoen et al.[15], which included using the dose-response model for the most infectious GI genogroup and Serogroup positive population (Se+), as well as assuming the entire population is susceptible.

Table 3 presents summary statistics of the pathogen concentrations computed over 10,000 Monte Carlo iterations.

Table 3. Pathogen occurrence (percentage of days per year with pathogens in the greywater), $\log_{10}$-value of arithmetic mean concentrations when occurring quantiles of concentrations (50% and 95%) when occurring, and geometric standard deviation (SD) when occurring in untreated greywater at three population sizes. CFU: colony-forming units; GC: genome copies. Adapted from Jahne et al.[1] with *E. coli* distributions from Sylvestre et al.[2].

**Reference pathogen**	**Unit**	**pop = 5**					**pop = 100**					**pop = 1000**				
	**$\log_{10}$#/L**	**Occ.**	**Mean**	**50%**	**95%**	**Geom. SD**	**Occ.**	**Mean**	**50%**	**95%**	**Geom. SD**	**Occ.**	**Mean**	**50%**	**95%**	**Geom. SD**
**Protozoa**																
Giardia spp.	cysts	0.7 %	4.2	1.8	3.9	1.2	11.5 %	3.1	0.5	2.6	1.2	69.8 %	2.3	0.0	1.9	1.2
Cryptosporidium spp.	oocysts	0.1 %	4.4	2.6	4.5	1-1	1.3 %	3.7	1.3	3.3	1.1	12.1 %	2.7	0.3	2.3	1.1
**Bacteria**																
Campylobacter spp.	CFU	0.2 %	4.6	1.9	4.7	1.5	3.2 %	4.4	0.6	3.4	1.5	27.4 %	3.2	-0.2	2.5	1.5
Salmonella spp.	CFU	0.2 %	4.6	2.8	4.8	1.1	2.7 %	3.7	1.5	3.4	1.1	23.7 %	2.8	0.6	2.5	1.1
**Viruses**																
Norovirus	GC	2.9 %	6.3	3.9	5.8	1.1	45.0 %	5.0	2.8	4.7	1.1	99.7 %	4.8	3.2	4.8	0.9
Adenovirus	particles	0.1 %	7.3	5.3	7.4	1.2	2.4 %	6.7	4.0	6.1	1.2	21.6 %	5.7	3.1	5.2	1.2

SI 2: Numeric effect of model simplification on LRTs

Eq. (8) is a conservative simplification, as it assumes that the risk under failure conditions is added to the baseline infection risk. A fully rigorous formulation would adjust the baseline ingestion volume by the volume ingested during a failure, and, for cases where $p_{use,failure} < 1$, include a correction term $p_{use,baseline,}$ with $p_{use,failure} + p_{use,baseline} = 1$. Figure 1 compares LRTs for a bimodal failure using the simplified model (A) and the rigorous model formulation (B), showing that LRTs obtained with the simplified model remain unchanged or increase by at most 0.1.

A. Simplified model

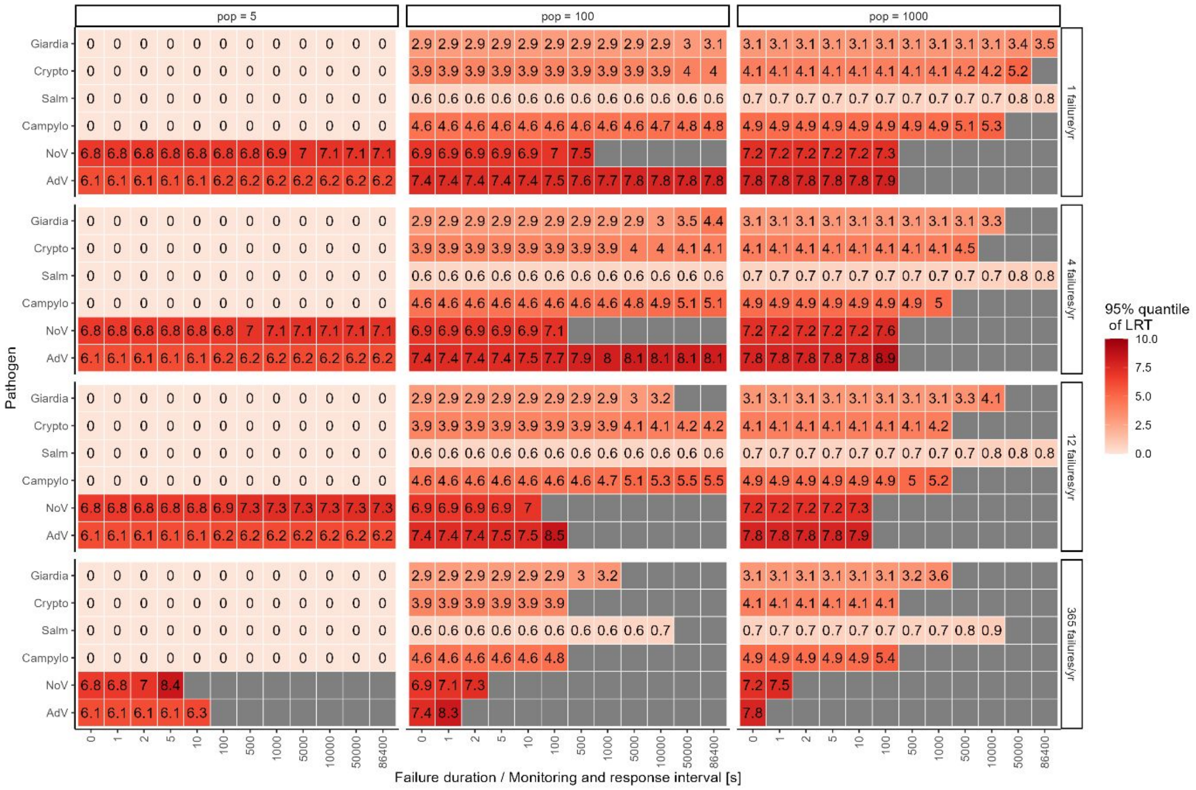

B. Model accounting for decrease in baseline infection risk

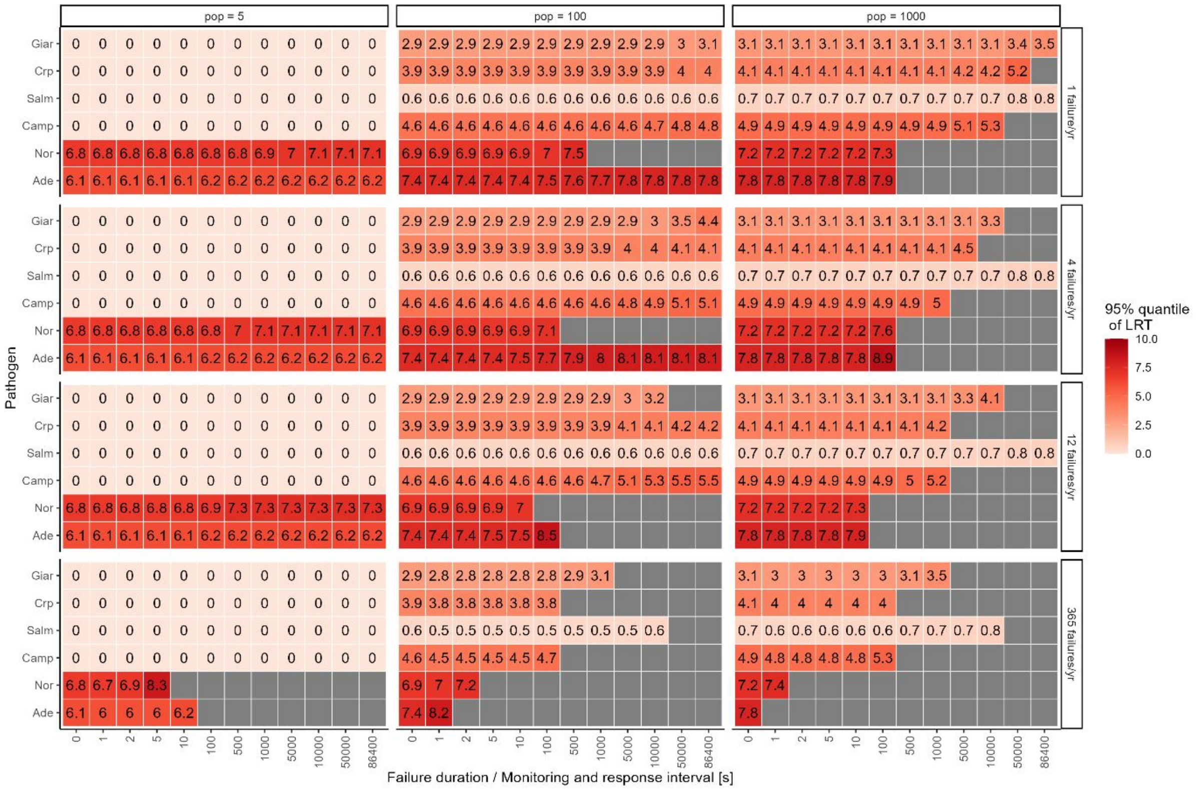

Figure 1. 95% quantiles of LRTs for the recycling of greywater at three scales for the complete failure of a bimodal failure for different failure durations (equivalent to the monitoring and response interval). Reference pathogens: Giardia: *Giardia* spp.; Crypto: *Cryptosporidium* spp.; Salm: *Salmonella* spp.; Campylo: *Campylobacter* spp.; NoV: norovirus; AdV: adenovirus. Grey boxes indicate that the health benchmark cannot be met for the respective failure duration. **A**: results for conservative simplified model. **B**: results for rigorous formulation accounting for the decrease in baseline infection risk.

SI 3: Results for all reference pathogens

SI 3.1: Log-removal targets without failure (baseline scenario)

In the absence of treatment failures, the LRTs for indoor reuse of treated greywater varied with scale (Figure 2). At a 5-people scale, the 95% quantiles of LRTs were zero for protozoa and bacteria, due to the low occurrence of these pathogens in such small populations. However, the LRTs for norovirus, the reference pathogen with the highest occurrence, requires similar levels of treatment independently of the collection scale. LRTs for adenovirus were also high at all scales, due to its high concentrations when occurring and the high the infectivity predicted by the applied dose-response model. Note that the LRTs are lower than those reported in Reynaert et al.[16], as the previous study included uses of reclaimed water with higher volumes of routine ingestion.

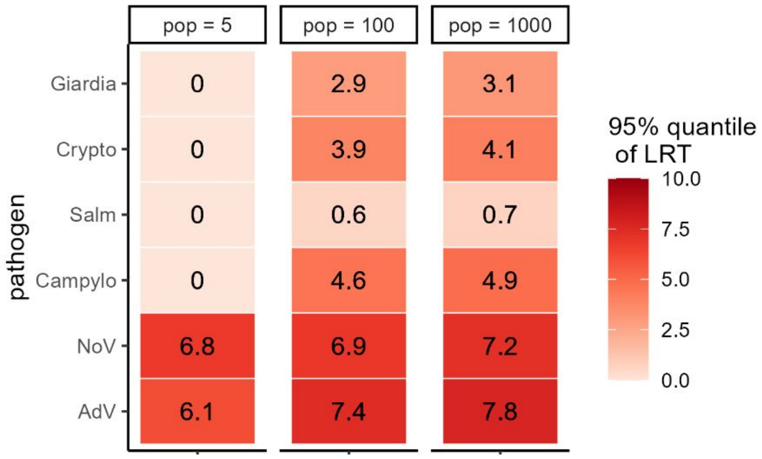

Figure 2. 95% quantiles of LRTs for the recycling of greywater at three scales in the absence of treatment failures. Reference pathogens: Giardia: *Giardia* spp.; Crypto: *Cryptosporidium* spp.; Salm: *Salmonella* spp.; Campylo: *Campylobacter* spp.; NoV: norovirus; AdV: adenovirus. Note that the 99% quantiles of LRTs were non-zero for the 5-people scale (LRTs of 3.4 for Giar, 3.8 for Crypto, 1.2 for Salm, and 5 for Campylo).

The 95% quantiles of LRTs did not vary by more than 0.1-$\log_{10}$ units between model runs, with the exception of adenovirus LRTs, which varied by up to 0.5-$\log_{10}$ units in the 5-people scenario. For adenovirus, the lack of a widely accepted dose-response model further adds to the model uncertainty. The generalized dose-response model from Teunis et al. [17] combines information from inhalation, oral ingestion and droplet inoculation. However, due to the limited data for oral ingestion, the model parameters are primarily influenced by the inhalation route. It is also limited to adenovirus types typically causing respiratory infections (adenovirus 4/7/16) with all oral challenge doses producing infection but no clinical symptoms. We adopted this model as it currently represents the best available information, but LRTs may need to be updated when dose-response models specific to oral ingestion of waterborne adenoviruses (types 40/41) become available.

SI 3.2: Failure of a bimodal treatment barrier

For a bimodal treatment failure, norovirus determined the minimum monitoring frequency in most scenarios, due to its relatively high prevalence (Figure 3). Only for the scenario with the highest (but unrealistic) failure frequency of 365 failures/year, adenovirus – the pathogen associated with the highest LRTs in the absence of failures in the larger-scale systems – requires higher minimum monitoring frequencies at the 100- and 1000-people scales

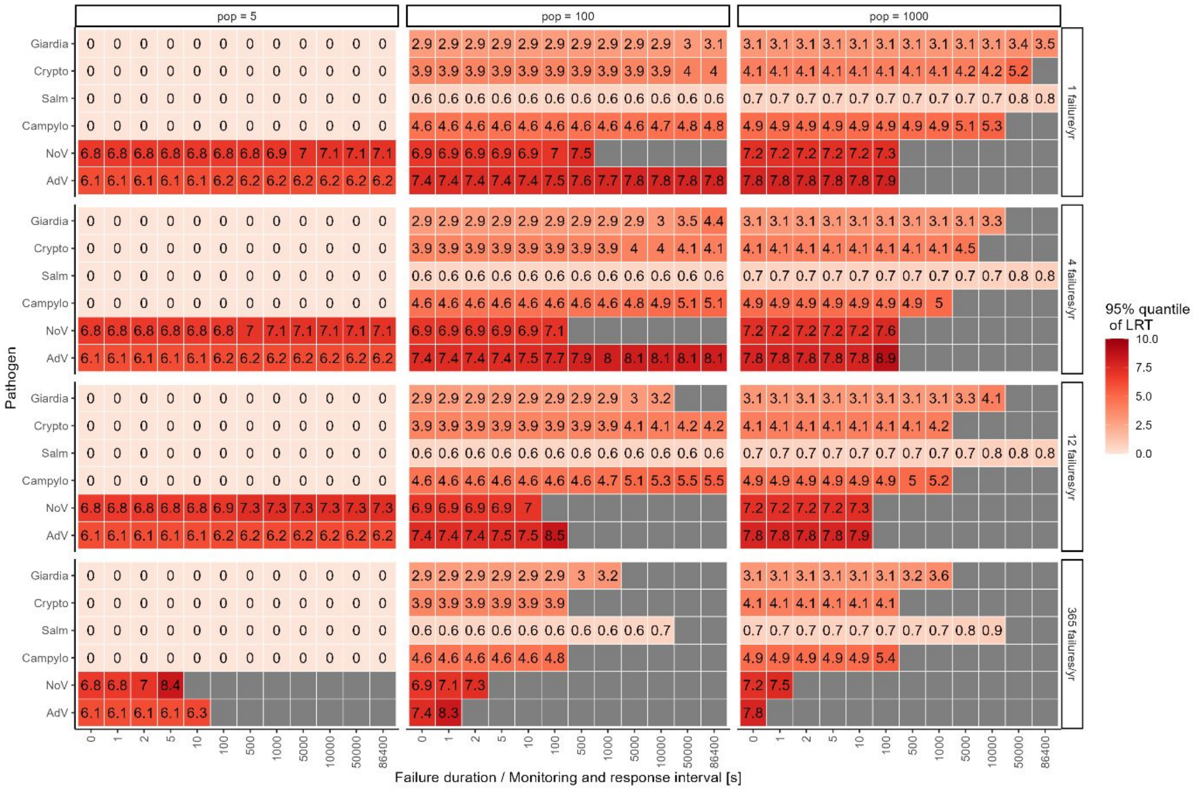

Figure 3. 95% quantiles of LRTs for the recycling of greywater at three scales for the complete failure of a bimodal failure for different failure durations (equivalent to the monitoring and response interval). Reference pathogens: Giardia: *Giardia* spp.; Crypto: *Cryptosporidium* spp.; Salm: *Salmonella* spp.; Campylo: *Campylobacter* spp.; NoV: norovirus; AdV: adenovirus. Grey boxes indicate that the health benchmark cannot be met for the respective failure duration.

SI 3.3: Failure of a chemical disinfectant barrier

For all reference pathogens – due to the presence of a disinfectant residual that buffers short-term treatment failures – the required monitoring intervals were longer than for bimodal treatment barriers, especially at higher failure frequencies (Figure 4).

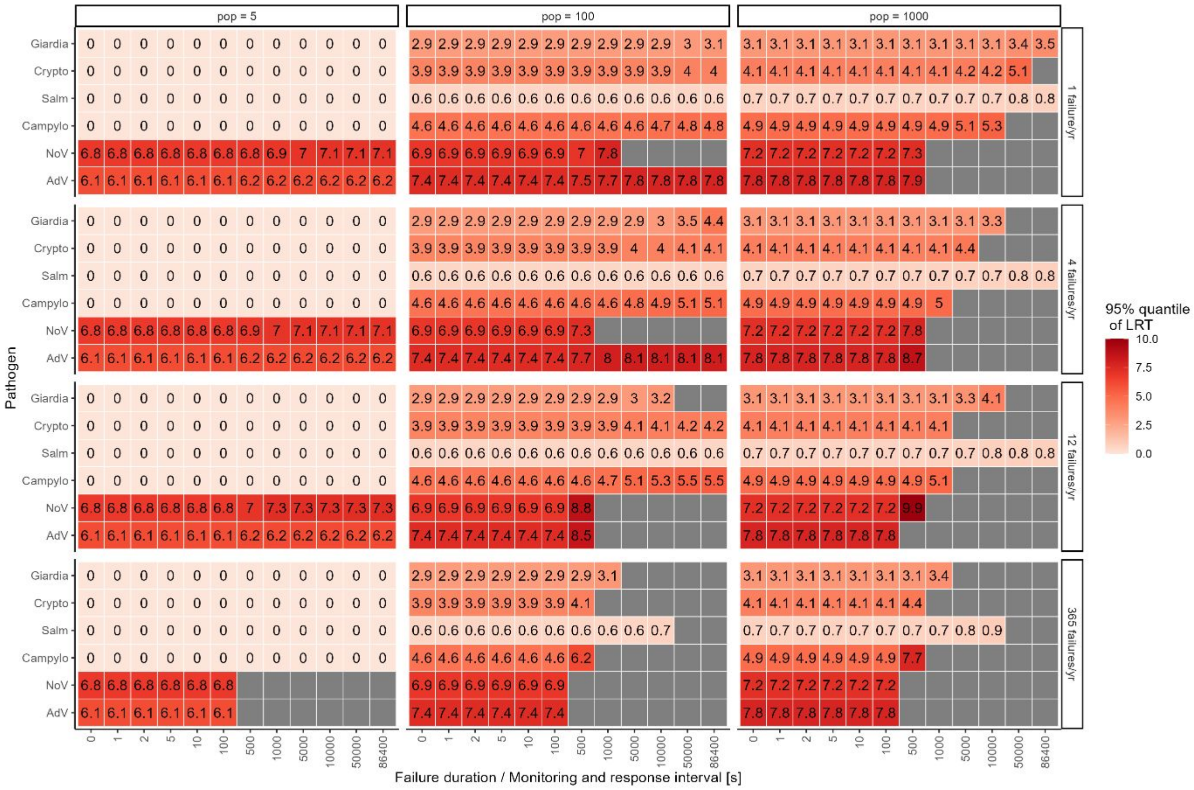

Figure 4. 95% quantiles of LRTs for the recycling of greywater at three scales for the complete failure of a chemical disinfectant barrier for different failure durations (equivalent to the monitoring and response interval). Reference pathogens: Giardia: *Giardia* spp.; Crypto: *Cryptosporidium* spp.; Salm: *Salmonella* spp.; Campylo: *Campylobacter* spp.; NoV: norovirus; AdV: adenovirus. Grey boxes indicate that the required LRT is equal to or larger than 10.

SI 4: Different allocations of LRVs between a bimodal barrier and a chemical disinfectant

Monitoring frequencies depended on the allocation of LRVs between the different treatment barriers (Figure 5).

a.) $LRV_{bimod} = 2$

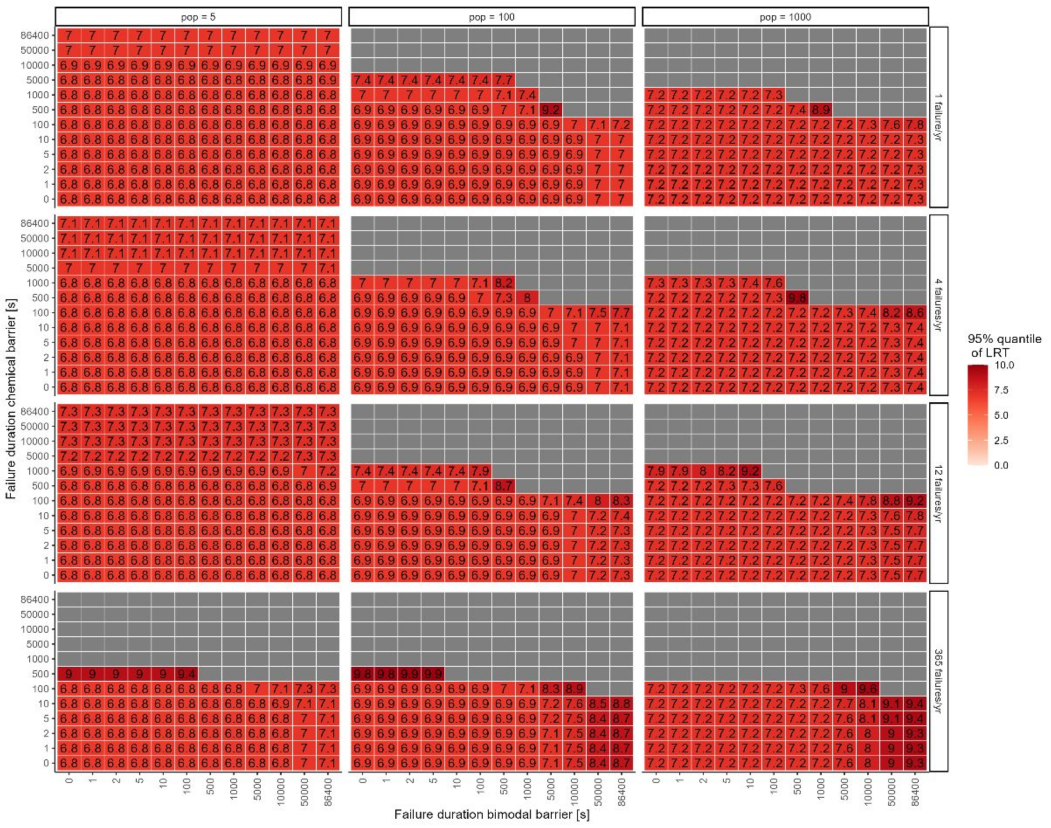

b.) $LRV_{bimod} = 3$
(main scenario)

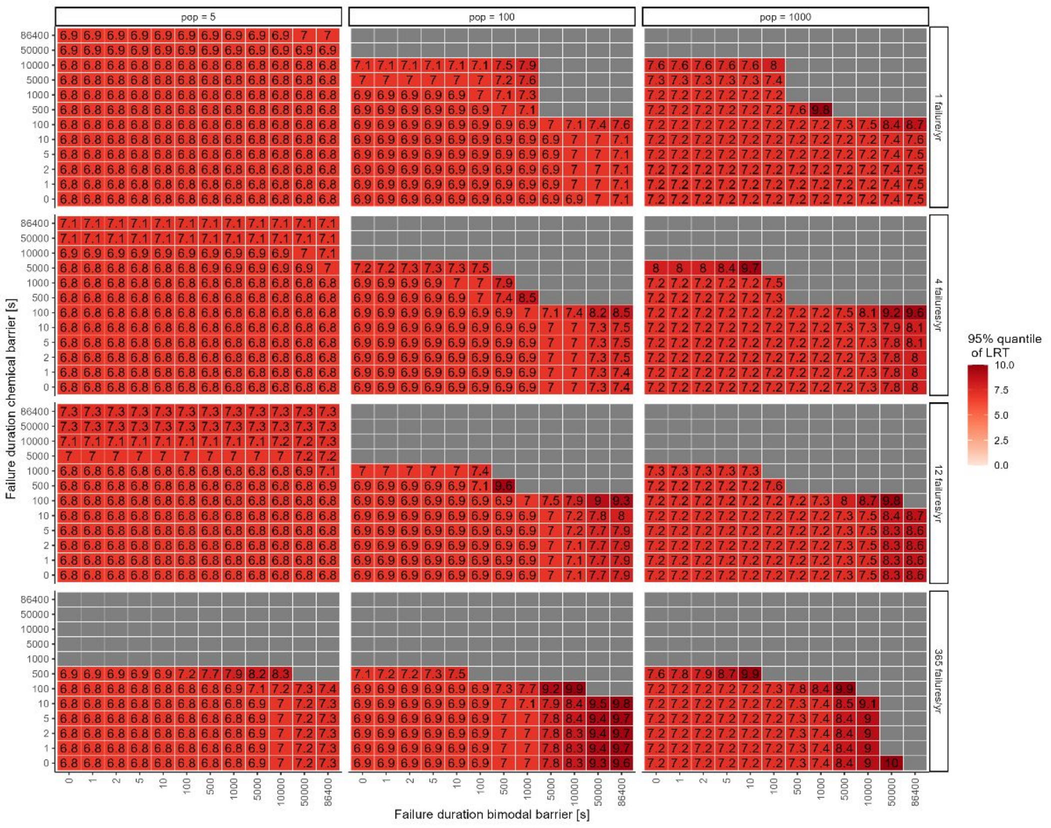

c.) $LRV_{bimod} = 4$

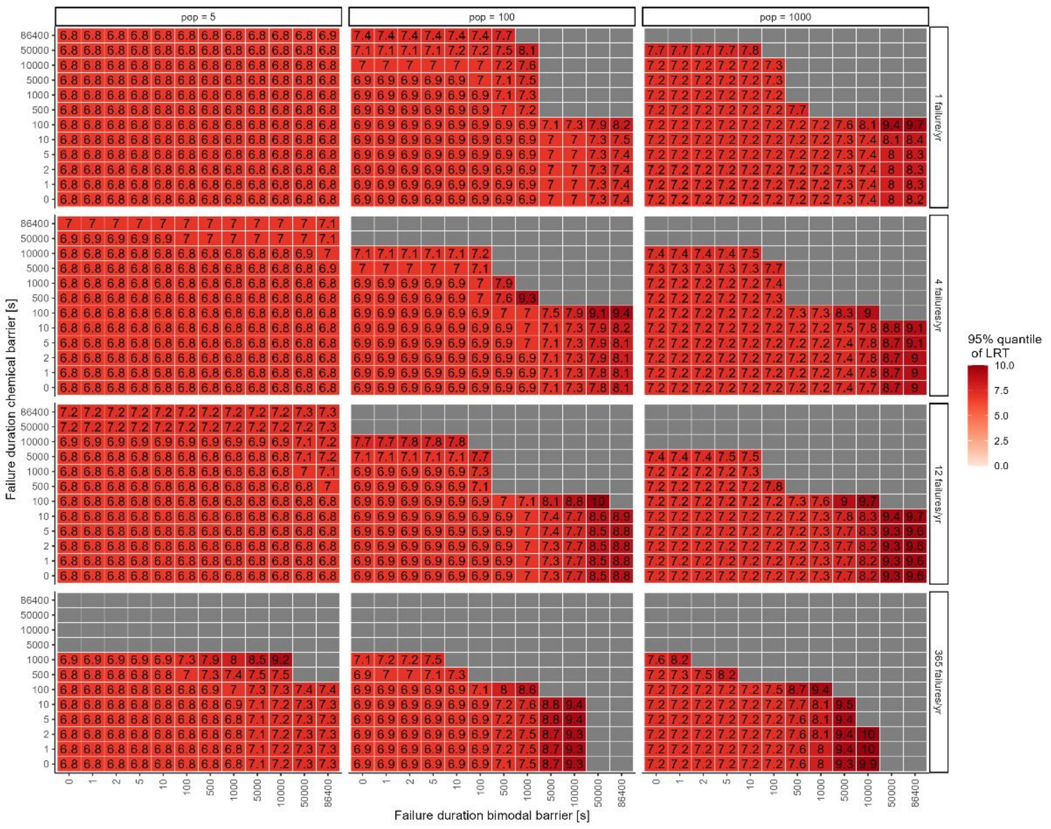

Figure 5. 95% quantiles of LRTs for norovirus for the recycling of greywater at three scales for the complete failure of a bimodal failure (contributing a fixed LRV_{bimod}) and a barrier with residual disinfectant (contributing the remaining required LRVs). Grey boxes indicate that the required LRT is equal to or larger than 10.

SI 5: Sensitivity analysis

The modeling results depend on several assumptions, including assumptions that are

- User-specific
    - Section 5.1: Use duration t_{use}
    - Section 5.2: Use frequency n_{use}
- System-specific
    - Section 5.3: HRT in the storage tank $HRT_{storage}$
- Treatment-specific
    - Section 5.4: HRT in the contact zone $HRT_{storage}$
    - Section 5.5: Hydraulics of the contact zone M

All presented modeling results are for a bimodal treatment barrier, with the exception of parameters specific to chemical disinfection (Sections 5.4 and 5.5).

SI 5.1 Dependency on t_{use}

a.) t_{use} = 30 s

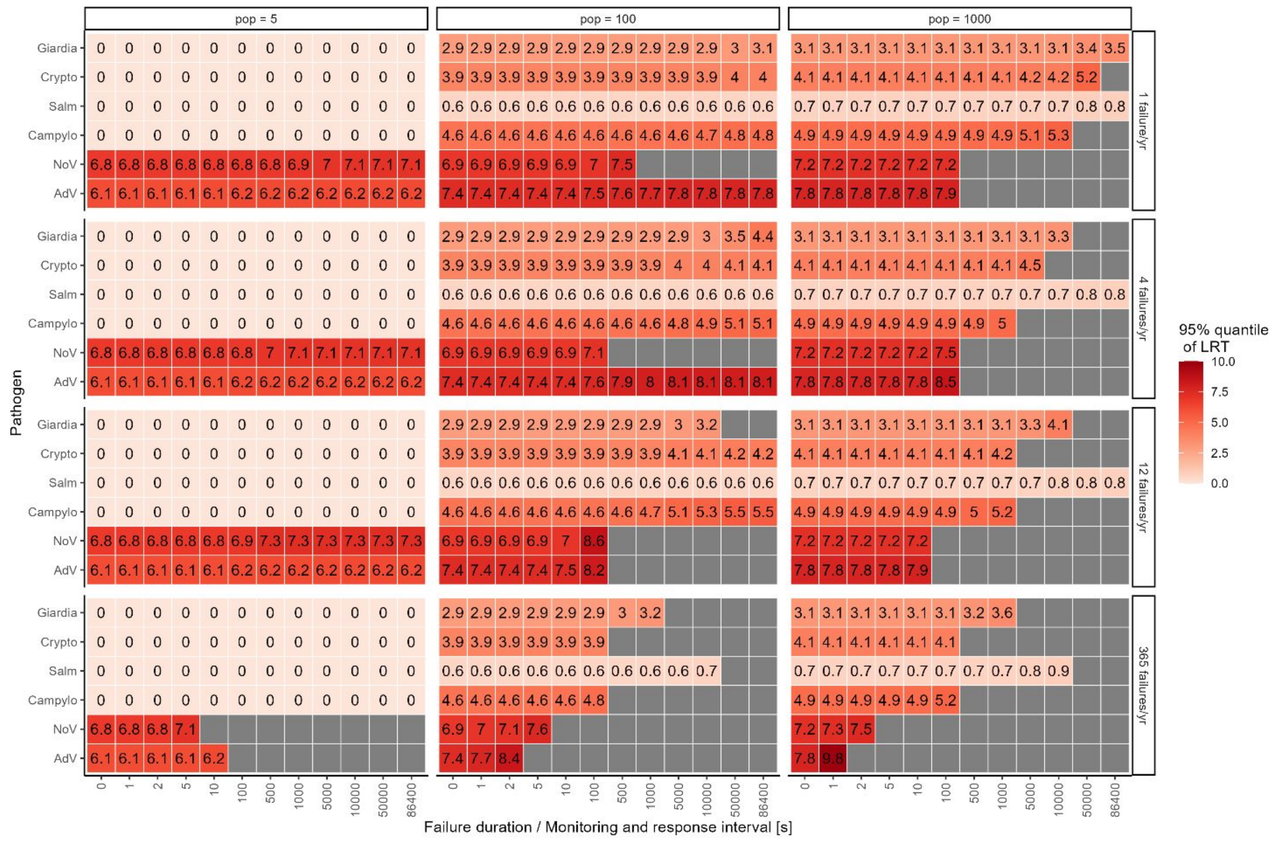

b.) t_{use} = 60 s
(main scenario)

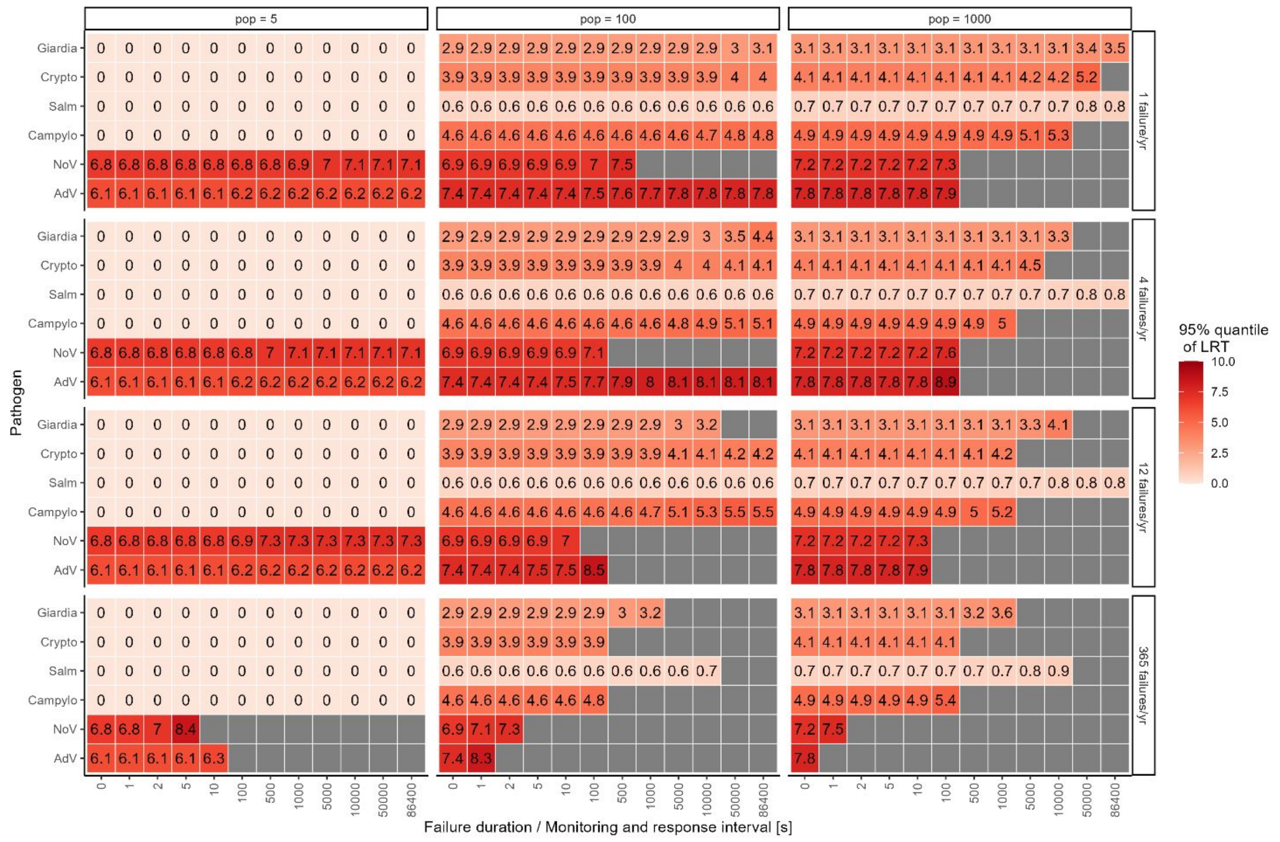

c) t_{use} = 120 s

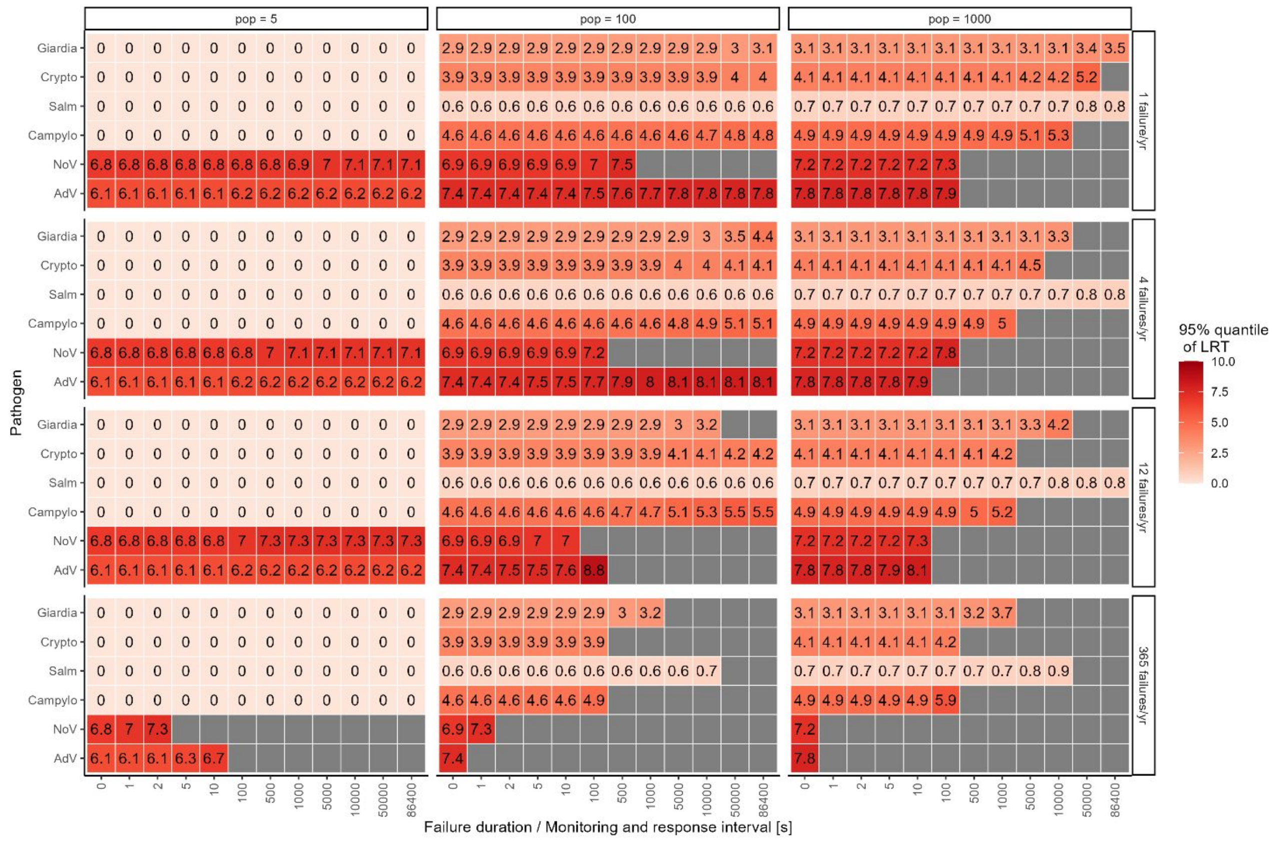

Figure 6. 95% quantiles of LRTs for the recycling of greywater at three scales for the complete failure of a bimodal failure for different failure durations (equivalent to the monitoring and response interval). Reference pathogens: Giardia: *Giardia* spp.; Crypto: *Cryptosporidium* spp.; Salm: *Salmonella* spp.; Campylo: *Campylobacter* spp.; NoV: norovirus; AdV: adenovirus. Grey boxes indicate that the health benchmark cannot be met for the respective failure duration.

SI 5.2 Dependency on n_{use}

a.) n_{use} = 2 uses / day

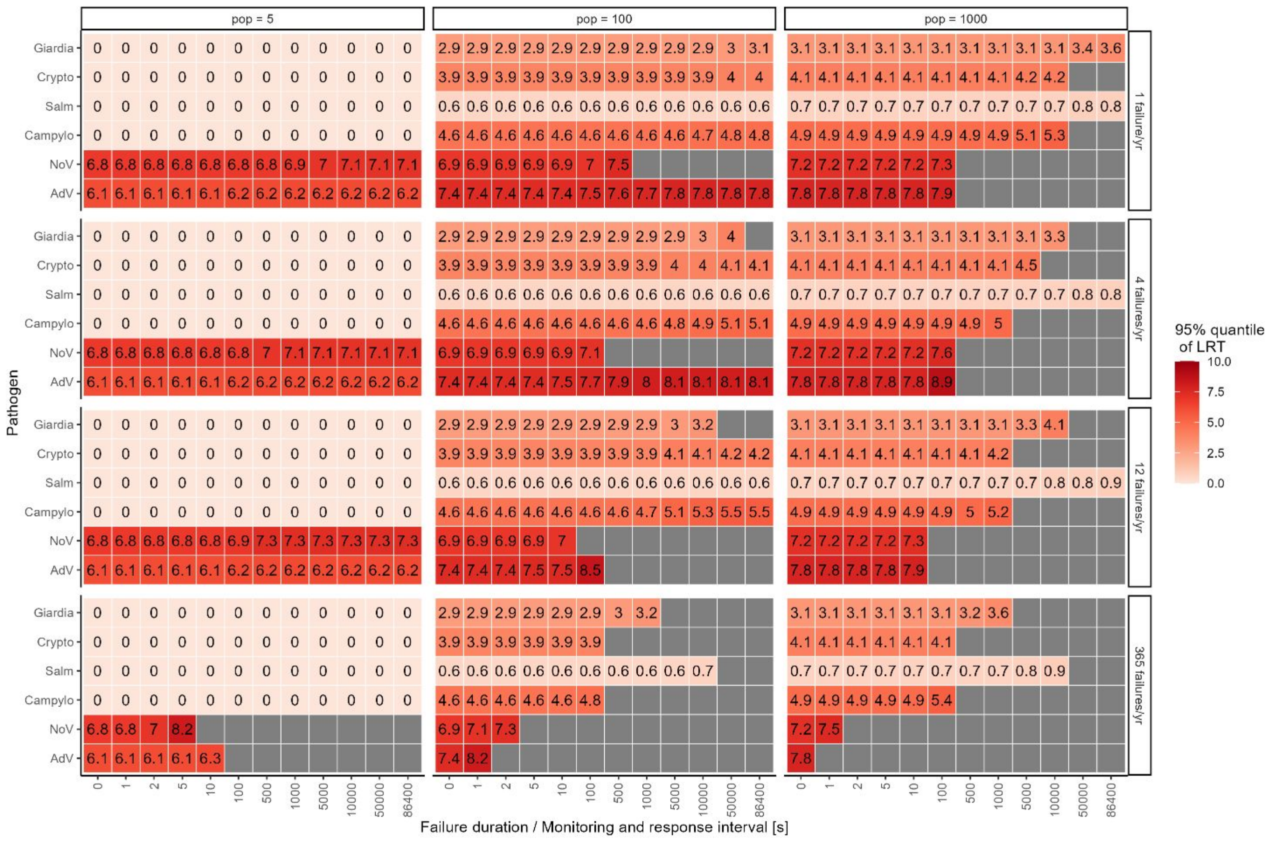

b.) n_{use} = 5 uses / day (main scenario)

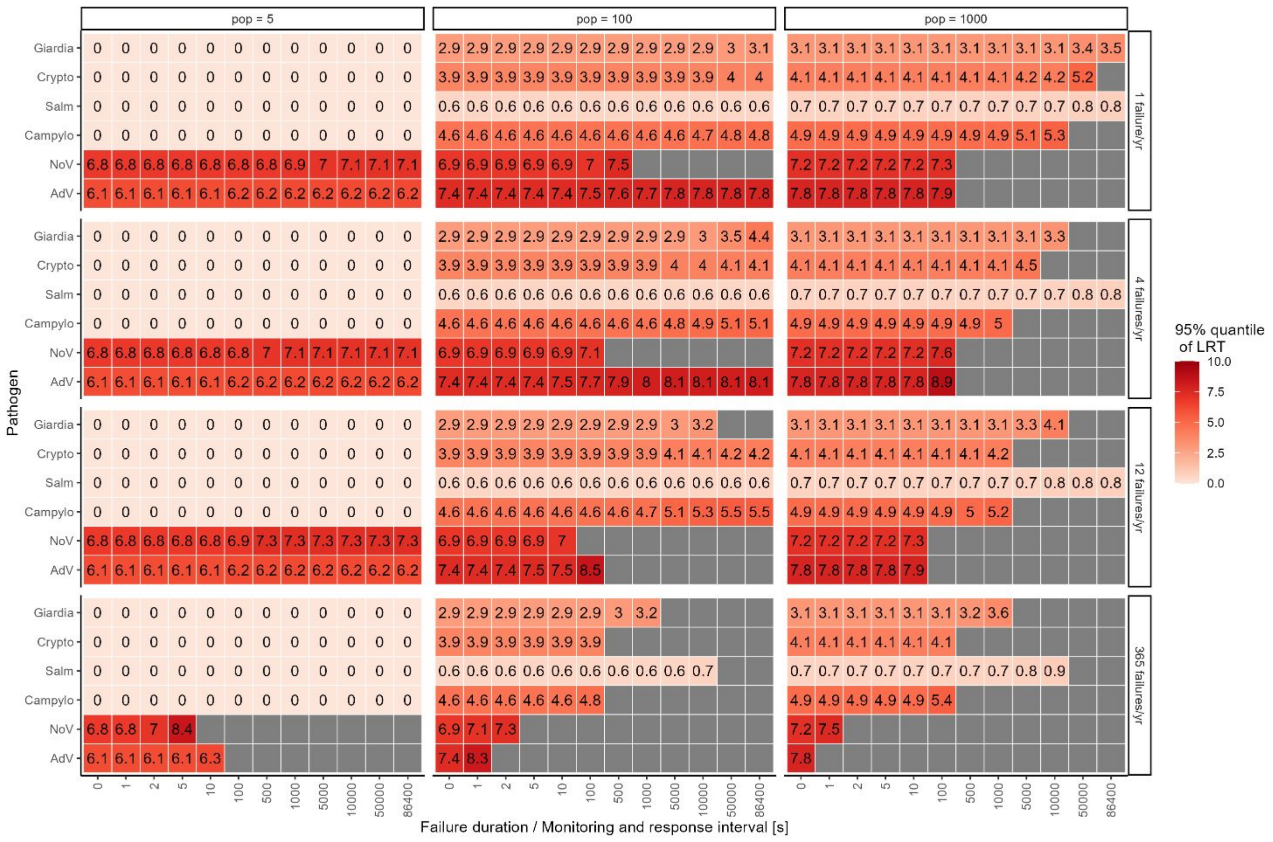

c.) n_{use} = 10 uses / day

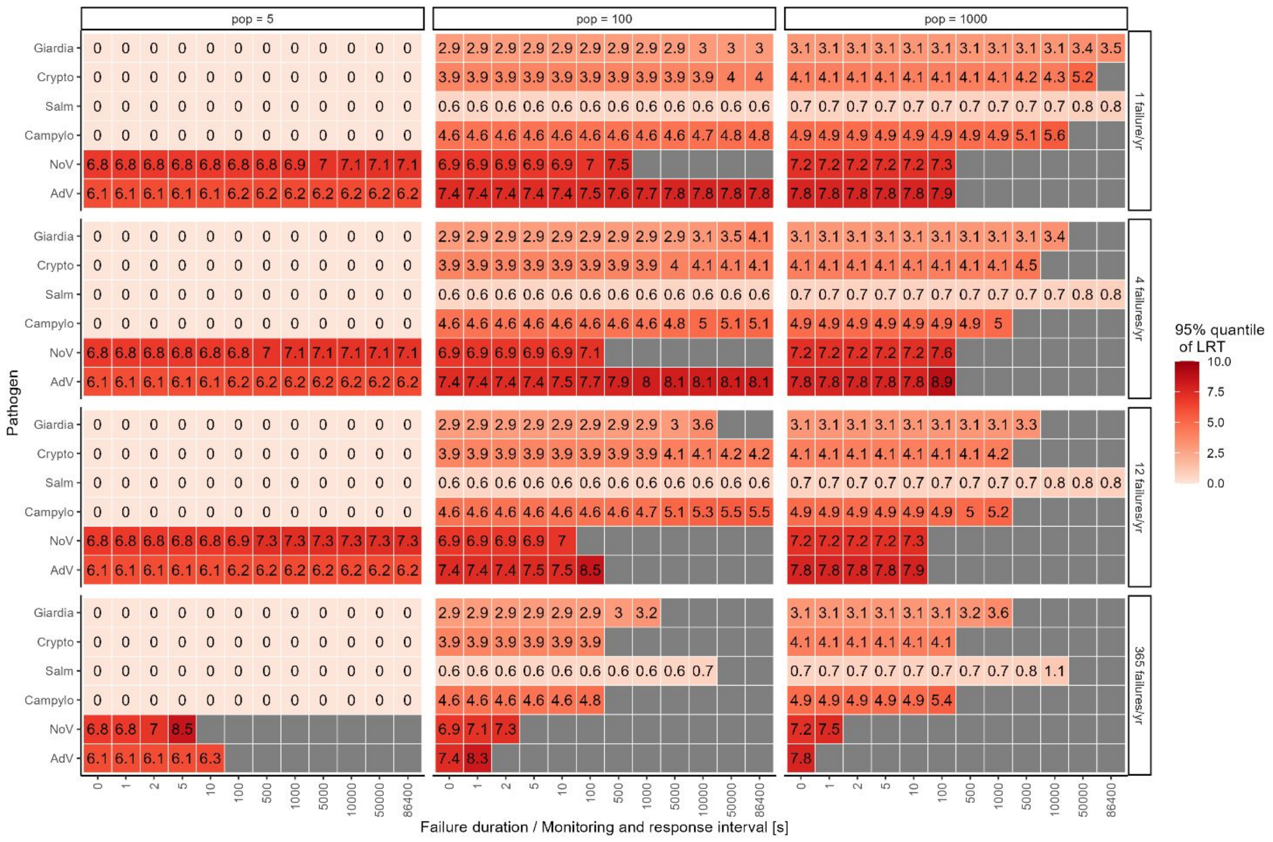

Figure 7. 95% quantiles of LRTs for the recycling of greywater at three scales for the complete failure of a bimodal failure for different failure durations (equivalent to the monitoring and response interval). Reference pathogens: Giardia: *Giardia* spp.; Crypto: *Cryptosporidium* spp.; Salm: *Salmonella* spp.; Campylo: *Campylobacter* spp.; NoV: norovirus; AdV: adenovirus. Grey boxes indicate that the health benchmark cannot be met for the respective failure duration.

SI 5.3 Dependency on $HRT_{storage}$

a.) $HRT_{storage}$ = 0 hours

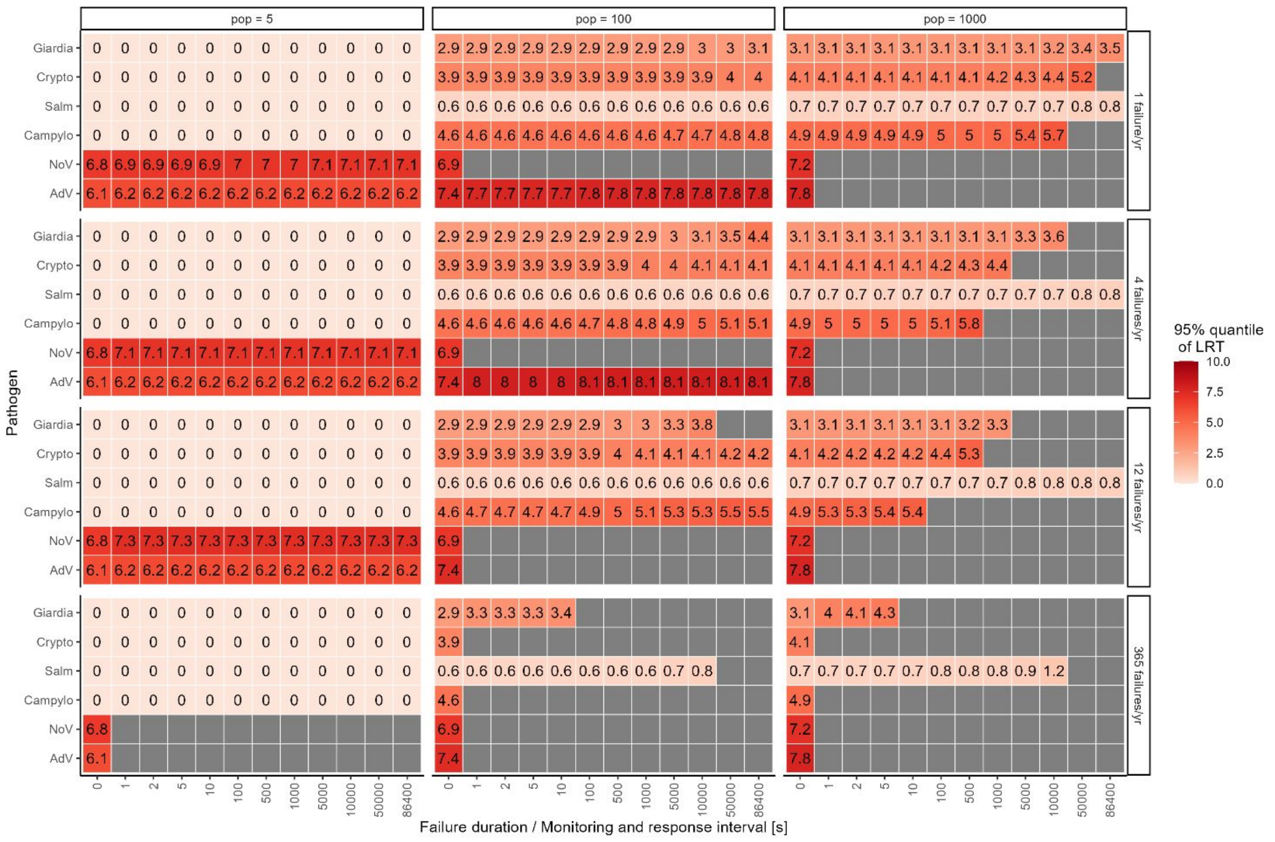

b.) $HRT_{storage}$ = 6 hours (main scenario)

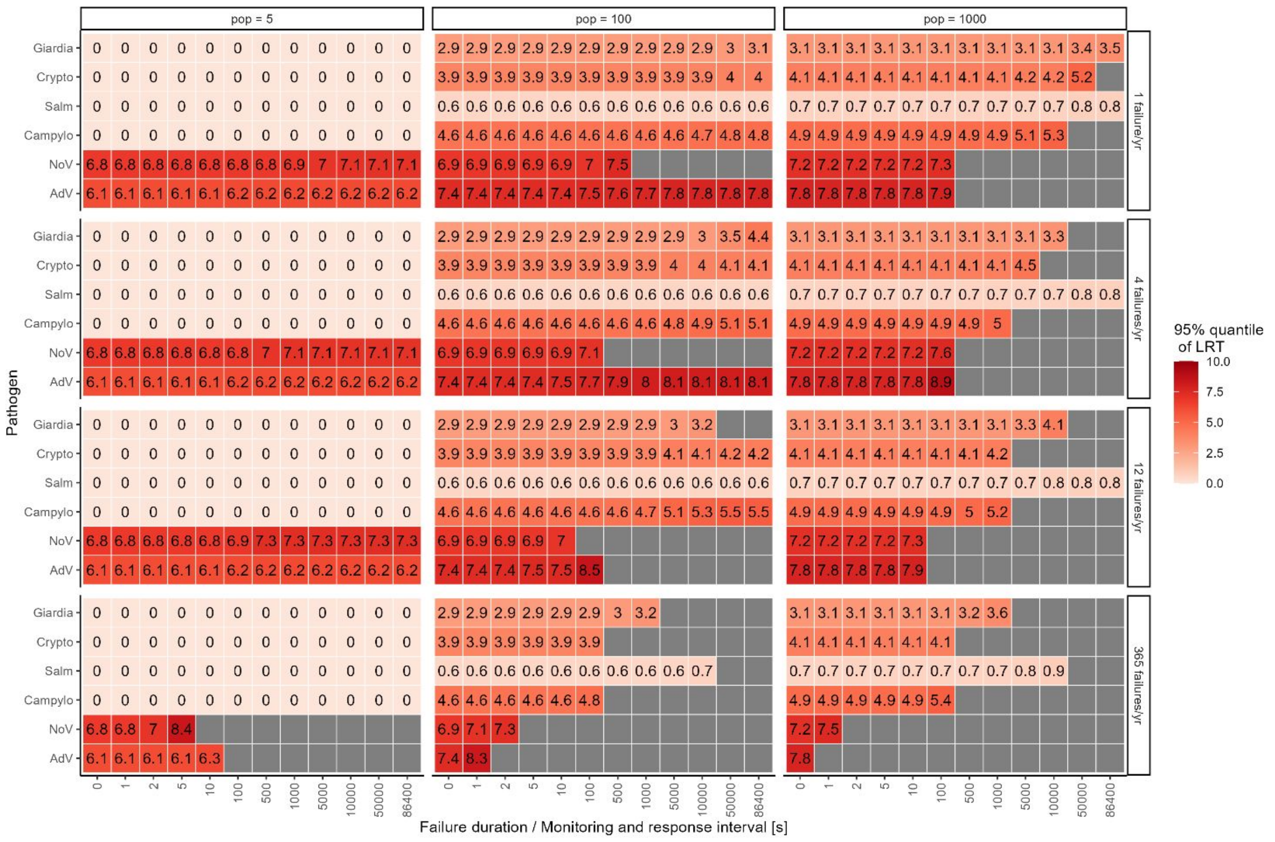

a.) $HRT_{storage}$ = 12 hours

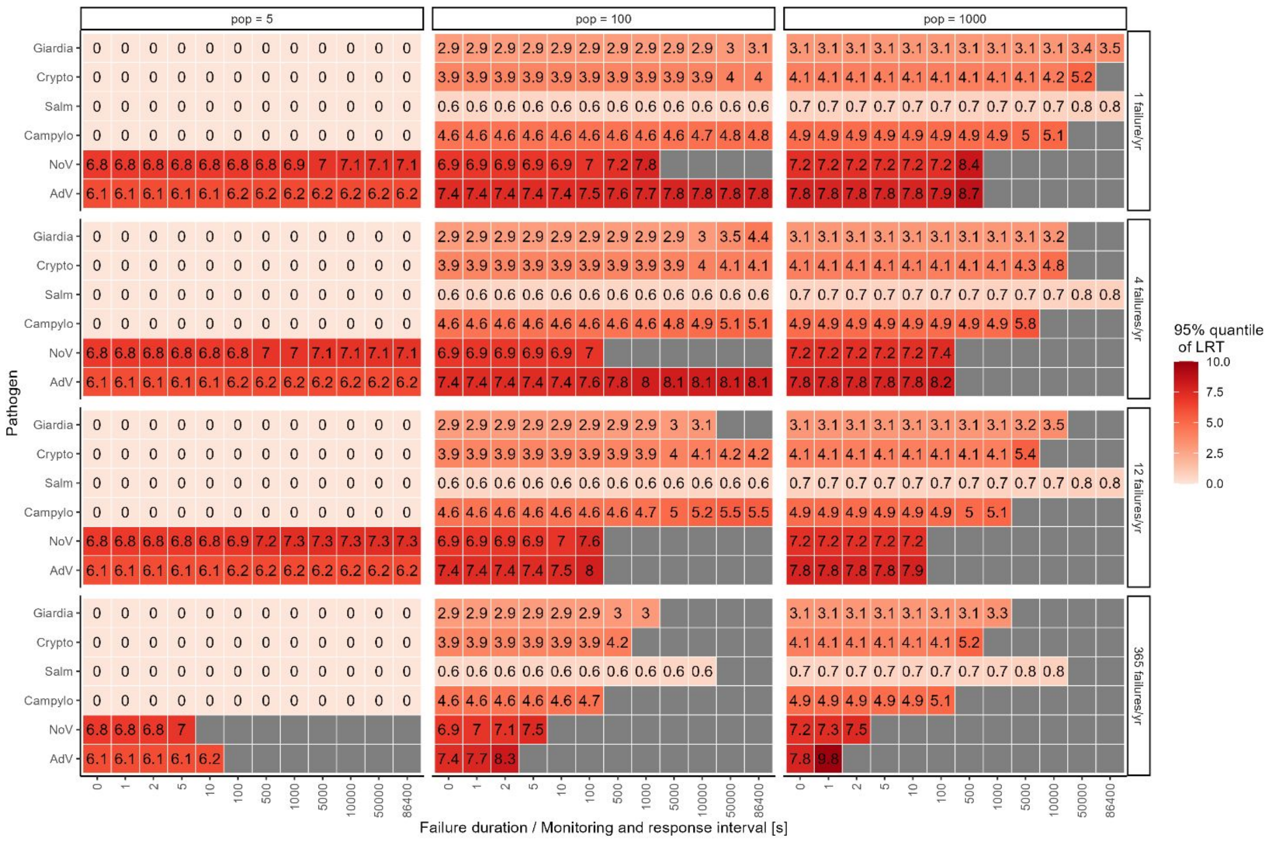

Figure 8. 95% quantiles of LRTs for the recycling of greywater at three scales for the complete failure of a bimodal failure for different failure durations (equivalent to the monitoring and response interval). Reference pathogens: Giardia: *Giardia* spp.; Crypto: *Cryptosporidium* spp.; Salm: *Salmonella* spp.; Campylo: *Campylobacter* spp.; NoV: norovirus; AdV: adenovirus. Grey boxes indicate that the health benchmark cannot be met for the respective failure duration.

SI 5.4 Dependency on $HRT_{contact}$

a.) $HRT_{contact}$ = 5 min

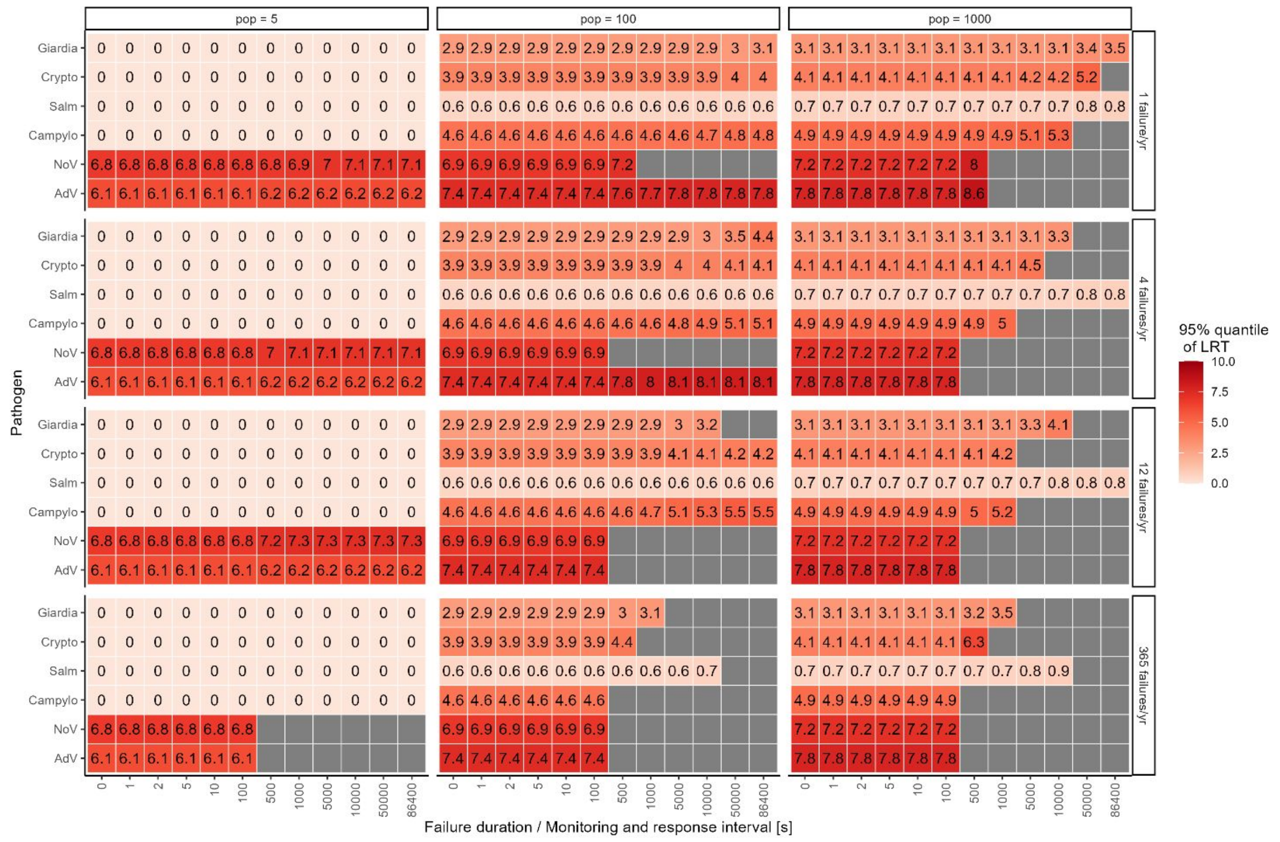

b.) $HRT_{contact}$ = 10 min (main scenario)

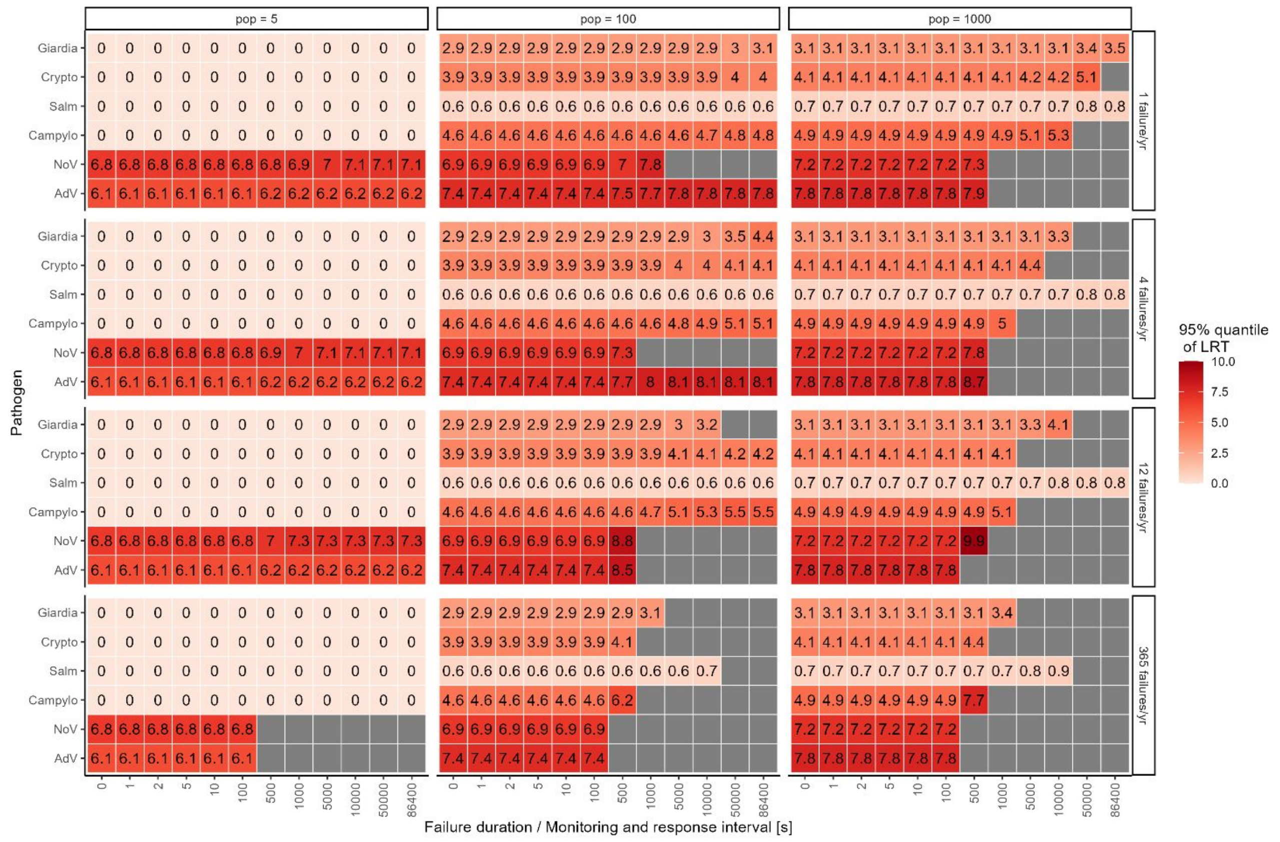

c.) $HRT_{contact}$ = 30 min

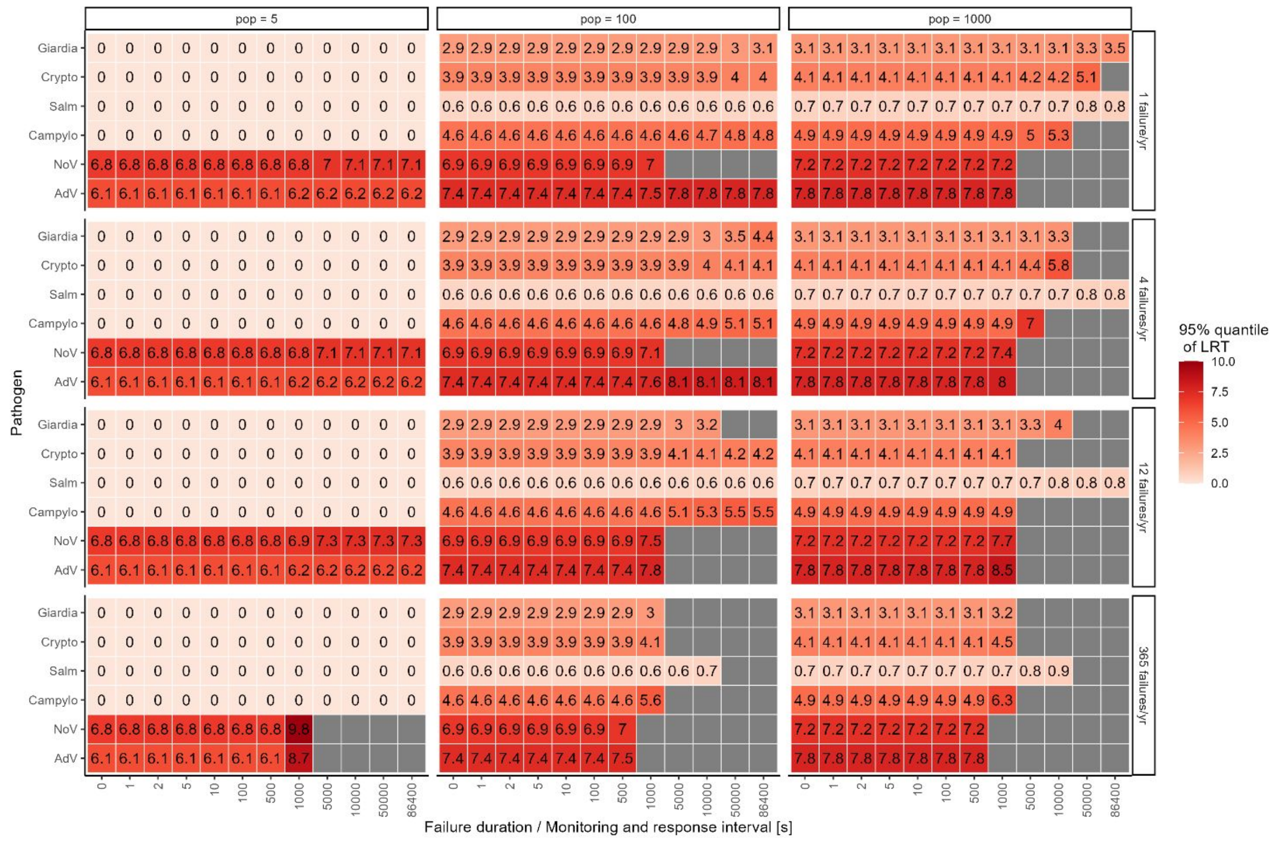

Figure 9. 95% quantiles of LRTs for the recycling of greywater at three scales for the failure of a chemical disinfectant barrier for different failure durations (equivalent to the monitoring and response interval). Reference pathogens: Giardia: *Giardia* spp.; Crypto: *Cryptosporidium* spp.; Salm: *Salmonella* spp.; Campylo: *Campylobacter* spp.; NoV: norovirus; AdV: adenovirus. Grey boxes indicate that LRT is equal to or larger than 10.

SI 5.5 Dependency on M

a.) M = 2
(poor hydraulics)

b.) M = 6
(main scenario)

c. M = 20
(near-perfect plug flow)

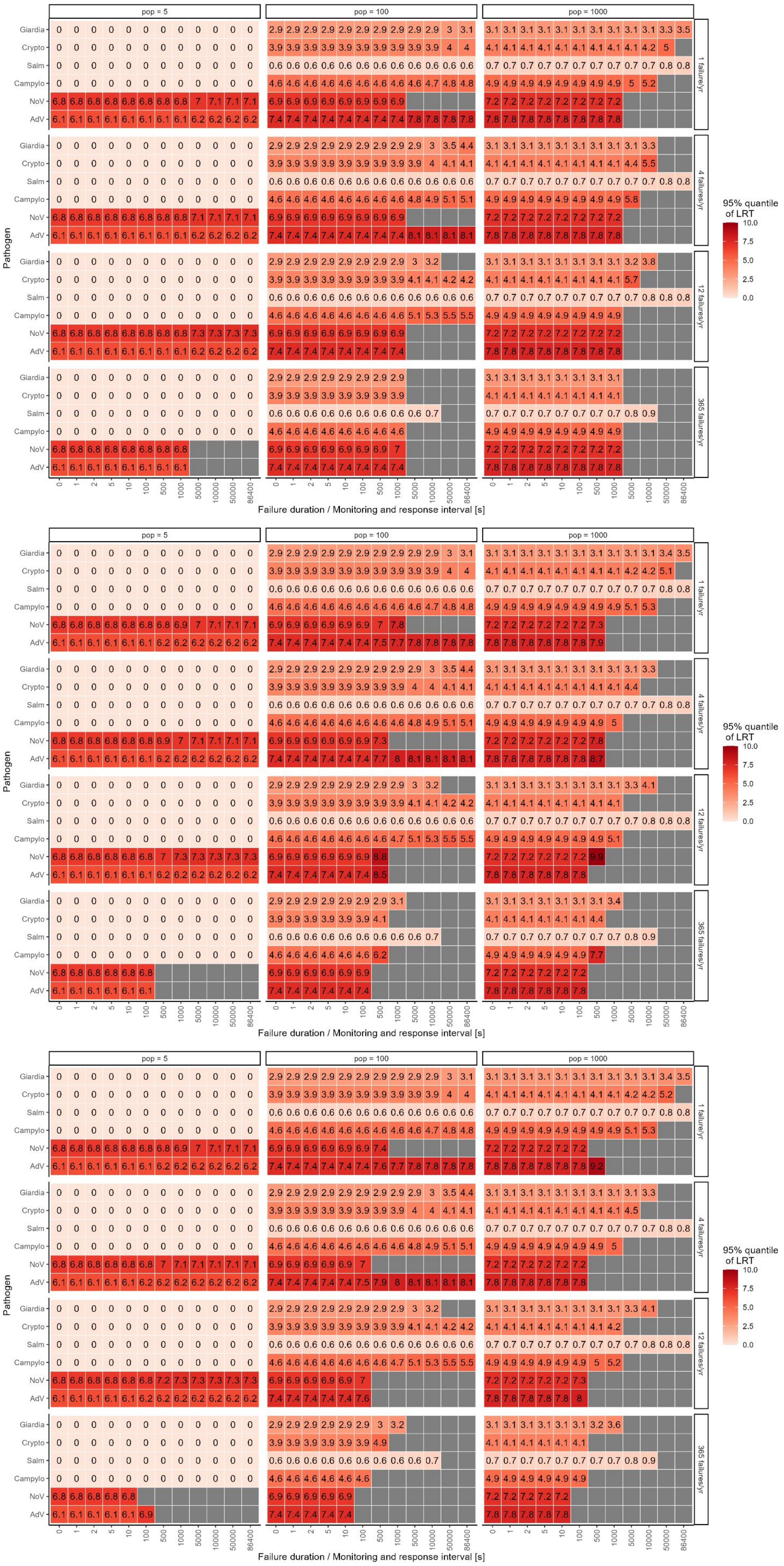

Figure 10. 95% quantiles of LRTs for the recycling of greywater at three scales for the failure of a chemical disinfectant barrier for different failure durations (equivalent to the monitoring and response interval). Reference pathogens: Giardia: *Giardia* spp.; Crypto: *Cryptosporidium* spp.; Salm: *Salmonella* spp.; Campylo: *Campylobacter* spp.; NoV: norovirus; AdV: adenovirus. Grey boxes indicate that LRT is equal to or larger than 10.

References

(1) Jahne, M. A.; Schoen, M. E.; Garland, J. L.; Ashbolt, N. J. Simulation of Enteric Pathogen Concentrations in Locally-Collected Greywater and Wastewater for Microbial Risk Assessments. *Microbial Risk Analysis* **2017**, *5*, 44–52.

(2) Sylvestre, É.; Jahne, M. A.; Reynaert, E.; Morgenroth, E.; Julian, T. R. A Critical Evaluation of Parametric Models for Predicting Faecal Indicator Bacteria Concentrations in Greywater. *Microbial Risk Analysis* **2024**, 100297.

(3) Scallan, E.; Hoekstra, R. M.; Angulo, F. J.; Tauxe, R. V.; Widdowson, M.-A.; Roy, S. L.; Jones, J. L.; Griffin, P. M. Foodborne Illness Acquired in the United States—Major Pathogens. *Emerging Infectious Diseases* **2011**, *17* (1), 7.

(4) Schönning, C.; Westrell, T.; Axel Stenström, T.; Arnbjerg-Nielsen, K.; Bernt Hasling, A.; Høibye, L.; Carlsen, A. Microbial Risk Assessment of Local Handling and Use of Human Faeces. *Journal of Water and Health* **2007**, *5* (1), 117–128.

(5) Rose, J. B.; Haas, C. N.; Regli, S. Risk Assessment and Control of Waterborne Giardiasis. *American Journal of Public Health* **1991**, *81* (6), 709–713.

(6) Petterson, S. R.; Mitchell, V. G.; Davies, C. M.; O'Connor, J.; Kaucner, C.; Roser, D.; Ashbolt, N. Evaluation of Three Full-Scale Stormwater Treatment Systems with Respect to Water Yield, Pathogen Removal Efficacy and Human Health Risk from Faecal Pathogens. *Science of the Total Environment* **2016**, *543*, 691–702.

(7) Messner, M. J.; Berger, P. Cryptosporidium Infection Risk: Results of New Dose-response Modeling. *Risk Analysis* **2016**, *36* (10), 1969–1982.

(8) Teunis, P. F. M.; Marinović, A. B.; Tribble, D. R.; Porter, C. K.; Swart, A. Acute Illness from Campylobacter Jejuni May Require High Doses While Infection Occurs at Low Doses. *Epidemics* **2018**, *24*, 1–20.

(9) Haas, C. N.; Rose, J. B.; Gerba, C. P. *Quantitative Microbial Risk Assessment*; John Wiley & Sons: Hoboken NJ, USA, 2014.

(10) Hall, A. J.; Rosenthal, M.; Gregoricus, N.; Greene, S. A.; Ferguson, J.; Henao, O. L.; Vinjé, J.; Lopman, B. A.; Parashar, U. D.; Widdowson, M.-A. Incidence of Acute Gastroenteritis and Role of Norovirus, Georgia, USA, 2004–2005. *Emerging Infectious Diseases* **2011**, *17* (8), 1381.

(11) Teunis, P. F. M.; Schijven, J.; Rutjes, S. A Generalized Dose-Response Relationship for Adenovirus Infection and Illness by Exposure Pathway. *Epidemiology & Infection* **2016**, *144* (16), 3461–3473.

(12) Atmar, R. L.; Opekun, A. R.; Gilger, M. A.; Estes, M. K.; Crawford, S. E.; Neill, F. H.; Graham, D. Y. Norwalk Virus Shedding after Experimental Human Infection. *Emerging Infectious Diseases* **2008**, *14* (10), 1553.

(13) Teunis, P. F. M.; Le Guyader, F. S.; Liu, P.; Ollivier, J.; Moe, C. L. Noroviruses Are Highly Infectious but There Is Strong Variation in Host Susceptibility and Virus Pathogenicity. *Epidemics* **2020**, *32*, 100401.

(14) Schoen, M. E.; Ashbolt, N. J.; Jahne, M. A.; Garland, J. Risk-Based Enteric Pathogen Reduction Targets for Non-Potable and Direct Potable Use of Roof Runoff, Stormwater, and Greywater. *Microbial Risk Analysis* **2017**, *5*, 32–43.

(15) Schoen, M. E.; Garland, J.; Soller, J. A.; Thimons, S. X.; Jahne, M. A. Onsite Nonpotable Water Systems Pathogen Treatment Targets: A Comparison of Infection and Disability-Adjusted Life Years (DALYs) Risk Benchmark Approaches. *Environmental Science & Technology* **2023**, *57* (26), 9559–9566.

(16) Reynaert, E.; Sylvestre, E.; Morgenroth, E.; Julian, T. R. Enteric Pathogen Log-Removal Targets and Treatment Trains for Greywater Recycling for Different Reuse Applications and Collection Scales. *Water Research* **2024**, 122216.

(17) Teunis, P. F. M.; Schijven, J.; Rutjes, S. A Generalized Dose-Response Relationship for Adenovirus Infection and Illness by Exposure Pathway. *Epidemiology & Infection* **2016**, *144* (16), 3461–3473.
